# Neural mechanism underlies CYLD modulation of morphology and synaptic function of medium spiny neurons in dorsolateral striatum

**DOI:** 10.3389/fnmol.2023.1107355

**Published:** 2023-02-08

**Authors:** Shu-yi Tan, Jin-xiang Jiang, Hui-xian Huang, Xiu-ping Mo, Jing-ru Feng, Yu Chen, Li Yang, Cheng Long

**Affiliations:** ^1^School of Life Sciences, South China Normal University, Guangzhou, China; ^2^School of Life Sciences, Guangzhou University, Guangzhou, China; ^3^South China Normal University-Panyu Central Hospital Joint Laboratory of Translational Medical Research, Panyu Central Hospital, Guangzhou, China

**Keywords:** CYLD, AMPAR, GluA1, GluA2, K63-linked ubiquitination, synaptic transmission, long-term depression, dorsolateral striatum

## Abstract

Although the deubiquitinase cylindromatosis (CYLD), an abundant protein in the postsynaptic density fraction, plays a crucial role in mediating the synaptic activity of the striatum, the precise molecular mechanism remains largely unclear. Here, using a *Cyld*-knockout mouse model, we demonstrate that CYLD regulates dorsolateral striatum (DLS) neuronal morphology, firing activity, excitatory synaptic transmission, and plasticity of striatal medium spiny neurons *via*, likely, interaction with glutamate receptor 1 (GluA1) and glutamate receptor 2 (GluA2), two key subunits of alpha-amino-3-hydroxy-5-methyl-4-isoxazole propionic acid receptors (AMPARs). CYLD deficiency reduces levels of GluA1 and GluA2 surface protein and increases K63-linked ubiquitination, resulting in functional impairments both in AMPAR-mediated excitatory postsynaptic currents and in AMPAR-dependent long-term depression. The results demonstrate a functional association of CYLD with AMPAR activity, which strengthens our understanding of the role of CYLD in striatal neuronal activity.

## 1. Introduction

Posttranslational protein modification by ubiquitin provides a plethora of distinct signals that have emerged as key regulators of neuronal activity, including postsynaptic function and plasticity (Mabb and Ehlers, 2010; Kantamneni et al., 2011). Ubiquitination by E3 ubiquitin ligases can be reversed by deubiquitinases (DUBs), while deregulation of DUBs has dramatic physiological consequences and causes a variety of diseases such as neurodegeneration or inflammatory disease (Palazon-Riquelme et al., 2018; Yang et al., 2021). Targeted deubiquitination *via* engineered DUBs corrects ion channelopathies caused by trafficking-deficient ion channels (Kanner et al., 2020).

CYLD belongs to a ubiquitin-specific protease family and specifically cleaves lysine 63-and methionine 1-linked polyubiquitin (polyUb) chains (Sato et al., 2015). CYLD was originally identified as a tumor suppressor in familial cylindromatosis, a skin tumor disorder, caused by *CYLD* mutations that lead to lack of DUB activity (Bignell et al., 2000). Although, CYLD is expressed at high levels in the brain (Mazarei et al., 2010), there have been surprisingly few studies of CYLD function in this vital organ.

Previous reports suggest that CYLD is enriched in the postsynaptic density (PSD) (Dosemeci et al., 2013; Ma et al., 2017) and regulates morphogenesis of dendrites and spines in hippocampal neurons (Li et al., 2019; Colombo et al., 2021). Recent studies indicate that the human *CYLD* gene, which maps to chromosome 16q12.1, is a causative gene for frontotemporal dementia (FTD) and amyotrophic lateral sclerosis: patients carrying *CYLD* mutations (*CYLD* p.Met719Val) display prominent memory impairment (Dobson-Stone et al., 2013, 2020; Tabuas-Pereira et al., 2020). The Met719Val variant is located in the DUB region of CYLD (amino acids 593–948) and increases K63-DUB activity. In addition, mouse hippocampal neurons transfected with CYLD_M719V_ show a significantly increased cytoplasmic localization of transactivator regulatory DNA-binding protein 43 (TDP-43) and decreased axonal length (Dobson-Stone et al., 2020). CYLD deficiency causes impaired fear memory, auditory neuropathy, and cognitive inflexibility (Li et al., 2021; Yang et al., 2021; Zajicek et al., 2022).

The *Cyld* gene is highly expressed in the dorsal striatum compared to 18 other brain regions (http://www.mouse.brain-map.org). The striatum, a brain region that is mainly (>95%) composed of GABAergic-projection medium spiny neurons (MSNs) (Mao et al., 2019), is implicated in neuropsychiatric diseases, such as Huntington’s disease and autism spectrum disorder (Mei et al., 2016; Crapser et al., 2020). A previous study showed that CYLD regulates striatal network function (Zhang et al., 2016), and plays an important role in striatal neuroinflammation; in addition, CYLD deficiency results in anxiety-like behavior (Han et al., 2020). Thus, the importance of CYLD in the striatum is well documented, but the molecular mechanism by which CYLD regulates synaptic function remains elusive.

MSNs receive excitatory synaptic inputs from cortical neurons; such corticostriatal MSN synapses can undergo long-term depression (LTD), which involves both activation of postsynaptic metabotropic glutamate receptors (mGluRs) and a requirement for presynaptic mechanisms that use endocannabinoids as retrograde messengers (Sung et al., 2001; Nazzaro et al., 2012). At excitatory synapses, mGluRs rapidly and selectively regulate synaptic efficacy by redistributing alpha-amino-3-hydroxy-5-methyl-4-isoxazole propionic acid receptors (AMPARs) on the spine surface (Luscher and Huber, 2010). AMPARs are tetrameric ion channels comprising four pore-forming homologous subunits (GluA1-GluA4), which mediate the majority of fast excitatory synaptic transmission (Yu et al., 2021; Zhang et al., 2021). All AMPAR subunits can be ubiquitinated, and K63-ubiquitin (K63Ub) chains are the primary posttranslational modification of GluA1 and GluA2, which map to K868 in GluA1 and K870/K882 in GluA2 (Widagdo et al., 2017). The ubiquitination of GluA1 and GluA2 facilitates AMPAR endocytosis, regulating the surface and synaptic expression of AMPARs (Lin et al., 2011; Huo et al., 2015). The trafficking, localization and stability of AMPARs are essential for regulating neuronal excitability and controlling synaptic plasticity in the brain (Beattie et al., 2000; Santos et al., 2009; Huganir and Nicoll, 2013; Henley and Wilkinson, 2016; Diering and Huganir, 2018). Although these studies address AMPAR-associated function, whether CYLD interacts with AMPAR to affect excitatory response of MSN has not been characterized.

Using electrophysiological, immunohistochemical and molecular biological approaches, we show in the present study that CYLD regulates GluA1 and GluA2 K63-linked ubiquitination, and CYLD deficiency impairs activity-dependent AMPAR removal. Furthermore, in line with these molecular data, we found that the absence of CYLD results in dramatic changes in MSN morphology, accompanied by decreased action potential (AP) firing, AMPAR-mediated excitatory postsynaptic activity, and LTD deficits in the corticostriatal pathway. Our results reveal a previously unsuspected role for CYLD in striatal excitatory synapses and illustrate a mechanism by which CYLD regulates neuronal morphology, excitability, and plasticity by regulating AMPAR stability and trafficking. These novel findings identify CYLD as a potential therapeutic target for the treatment of striatal functional abnormalities.

## 2. Materials and methods

### 2.1. Animals

All experimental procedures were approved by the South China Normal University Animal Care and Use Committee. *Cyld^+/−^* mice were generously provided by Dr. Shao-cong Sun (University of Texas MD Anderson Cancer Center, Texas, USA). In all experiments, both male and female 3-to 5-month-old *Cyld^+/+^* mice and their *Cyld^−/−^* littermates, generated from *Cyld^+/−^* mice, were used according to international and university ethical standards. Genotyping was performed by PCR, as previously described (Reiley et al., 2007), using tail DNA and the following primers: *Cyld* forward primer CCAGGCACTTTGAATTGCTGTC; *Cyld* reverse primer 1 CGTTCTTCCCAGTAGGGTGAAG; *Cyld* reverse primer 2 GCATGCTCCAGACTGCCTTGG. Animals were given *ad libitum* access to food and water and housed on a 12 h light/dark cycle at 22–25°C.

### 2.2. Cell culture and transfection

HEK293 cells were cultured in Dulbecco’s Modified Eagle Medium supplemented with 10% fetal bovine serum. HEK293 cells were transfected with the indicated plasmids with calcium phosphate (Clontech). At 48 h after transfection, HEK293 cells were washed with ice-cold phosphate buffered saline (PBS), centrifuged at 5000 rpm at 4°C for 10 min, and the precipitate was collected and homogenized using ice-cold sodium dodecyl sulphate (SDS) lysis buffer containing protease inhibitors (Beyotime, China) and phosphatase inhibitors (Beyotime, China), and then centrifuged at 14000 rpm for 10 min at 4°C.

### 2.3. Western blotting

Mouse brains were dissected on ice and the DLS tissues were separately sectioned in a cryostat as previously described (Peng et al., 2021). Tissues were homogenized in ice-cold lysis buffer containing (in mM) 50 Tris (pH 7.5), 5 EDTA, 150 NaCl, 1% SDS, protease inhibitors and phosphatase inhibitors and kept at 4°C for 40 min before cellular debris was removed by centrifugation at 14000 rpm for 10 min at 4°C. The supernatant was collected, its protein concentration determined and subsequently denatured for 20 min at 75°C. To extract cell membrane proteins, we followed the manufacturer’s instructions for the Membrane and Cytosol Protein Extraction Kit (P0033, Beyotime, China) (Li et al., 2018; Jiang et al., 2022). Briefly, DLS membrane proteins were extracted at 0–4°C using 1 ml solution A from the kit containing complete a proteinase and phosphatase inhibitor cocktail (Complete Mini; Roche) with 1 min homogenization. Cellular debris and nucleus were removed by centrifugation at 700× *g* for 10 min at 4°C, and then cell surface debris were collected by centrifugation at 14,000× *g* for 30 min at 4°C. The DLS membrane proteins were extracted by adding 200 μl solution B and incubating for 10 min at 4°C, and then centrifuging at 14,000× *g* for 5 min at 4°C. The supernatant was collected for denaturation at 75°C for 20 min. The protein samples were either stored or mixed with 25% (by volume) 5× SDS loading buffer at 75°C for 20 min prior to sodium dodecyl sulfate-polyacrylamide gel electrophoresis (SDS-PAGE), then electrotransferred onto a nitrocellulose membrane. The membrane was blocked with 5% non-fat dry milk in Tris-buffered saline (TBS) containing 0.5% Tween-20 (TBST) for 1 h at room temperature (RT) and incubated overnight at 4°C with the appropriate primary antibodies in TBST; anti-β-actin (AF0003, Beyotime, China), anti-α-tubulin (AF0001, Beyotime, China) or anti-sodium potassium ATPase (AF1864, Beyotime, China) was used as loading control (see Table 1 for details). Following washing and incubation with suitable secondary antibodies for 1 h at RT, and three 10-min washes with TBST, protein bands were visualized using an Immobilon Western ECL system (Bio-Rad, USA) and analyzed with Gel-Pro Analysis software (Media Cybernetics, USA).

**Table 1:** Key reagents and resources used in the present study.

Reagent or resource	Source	Identifier
*Plasmids*		
Prk5-HA-Ubiquitin-K63	Addgene	#17606
CYLD	Sino Biological	HG17235-UT
C-Myc-GRIA1	Sino Biological	HG15792-CM
C-Myc-GRIA2	Sino Biological	MG57202-CM
*Antibodies*		
mouse anti-Myc tag	Abcam	Ab32
mouse anti-GluA1	Synaptic Systems	182,011
rabbit anti-GluA1	Abcam	ab31232
rabbit anti-GluA2	Abcam	ab206293
mouse anti-GluA2	Santa Cruz	sc-517,265
mouse anti-GluA1-NTD	Sigma	MAB2263
mouse anti-GluA2-NTD	Sigma	MAB397
rabbit anti-CYLD	Proteintech	11,110-1-AP
mouse anti-CYLD	Santa Cruz	SC-74435
rabbit anti-CaMKII	Abcam	ab52476
rabbit anti-CaMKII (phosphor T286)	Abcam	ab32678
rabbit anti-Ub-K63	Millipore	05–1,308
mouse anti-Ub-K63	Millipore	05–1,313
rabbit anti-mGluR5	Millipore	AB5675
rabbit anti-NMDAR1	Abcam	ab109182
rabbit anti-NMDAR2B	Abcam	ab65783
rabbit anti-α-tubulin	Beyotime	AF0001
rabbit anti-β-actin	Beyotime	AF0003
rabbit anti-sodium potassium ATPase	Beyotime	AF1864
*Chemicals, peptides, and recombinant proteins*		
Picrotoxin	Sigma	P-1675
DHPG	Tocris Bioscience	0342
QX-314	Calbiochem	552,233
Biocytin	Sigma	B4261
Streptavidin Alexa 488	Invitrogen	S11223
Alexa Fluor 594 anti-mouse	Invitrogen	A21203
Protein A + G Agarose	Beyotime	P2012
Membrane and Cytosol Protein Extraction Kit	Beyotime	P0033
c-Myc Peptide	Beyotime	P9805
TCEP	Beyotime	ST045
*Software and algorithms*		
pCLAMP10.4	Molecular Devices	RRID: SCR_011323
ClampFit10.4	Molecular Devices	N/A
MiniAnalysis	Synaptosoft	https://minianalysis.software.informer.com/
ImageJ	NIH	https://imagej.net/software/fiji/downloads
ZEN software	Zeiss	https://www.zeiss.com
GraphPad Prism 8.0.2	GraphPad	https://www.graphpad.com/
Gel-Pro Analysis software	Media Cybernetics	https://www.mediacy.com/
SPSS	IBM	https://www.ibm.com/analytics/spss-statistics-software

### 2.4. Co-immunoprecipitation

Mouse brain tissue and cell lysates were obtained and prepared as previously described (Chen et al., 2017). Lysates were incubated with 5 μg CYLD antibody (sc-74,435, Santa Cruz, USA), GluA1 antibody (182,011, Synaptic Systems, USA), GluA2 antibody (ab206293, Abcam, UK) or Myc antibody (ab32, Abcam, UK) (or IgG as a control) overnight at 4°C with rotation. Protein A + G agarose (P2012, Beyotime, China) was added to each sample at 4°C for 3–4 h. Subsequently, three 5-min washes were made with cold lysis buffer. The precipitates were eluted from the beads by adding the same volume of 2 × SDS loading buffer and heating for 20 min at 75°C. Then, the samples were used for western blotting as described above.

### 2.5. *In vitro* deubiquitination assay

The *in vitro* deubiquitination assay was performed as described previously (Huo et al., 2015; Ma et al., 2017). GluA1 and GluA2 were purified from denatured lystes of HEK293 cells overexpressing Myc-GluA1 or Myc-GluA2 and HA-K63Ub by immunoprecipitation with anti-Myc and elution with Myc peptide (P9805, Beyotime, China). CYLD was purified from lysates of HEK293 cells overexpressing CYLD. Deubiquitination reactions were performed in deubiquitination buffer (in mM) (40 Tris, pH 7.1, 100 NaCl, 4 Tris (2-carboxyethyl) phosphine hydrochloride and 25% glycerol). Myc-GluA1 with K63Ub was incubated with or without CYLD for 2 h at 37°C. 1× SDS loading buffer was then added and the samples were heated for 20 min at 75°C prior to western blotting.

### 2.6. Acute brain slice preparation

Mice were anesthetized with chloral hydrate and transcardially perfused with prechilled and oxygenated modified artificial cerebrospinal fluid (aCSF) containing the following (in mM): 93 N-methyl-D-glucamine, 93 HCl, 2.5 KCl, 1.2 Na_2_HPO_4_, 30 NaHCO_3_, 20 HEPES, 25 D-glucose, 5 sodium L-ascorbate, 2 thiourea, 3 sodium pyruvate, 10 MgSO_4_, 0.5 CaCl_2_ (pH 7.3) saturated with 95% O_2_/5% CO_2_. Coronal 320 μm striatal slices were cut using a vibratome (VT1000S, Leica, Germany). Slices containing the cortex and striatum were continuously bathed in incubation aCSF (in mM): 92 NaCl, 2.5 KCl, 1.2 NaH_2_PO_4_, 30 NaHCO_3_, 20 HEPES, 25 D-glucose, 5 sodium L-ascorbate, 2 thiourea, 3 sodium pyruvate, 2 CaCl_2_, and 2 MgSO_4_ (pH 7.3). For electrophysiology recordings, the slices were recovered at RT for at least 1 h before a single slice was transferred to a submersion chamber perfused with 95% O_2_/5% CO_2_-saturated recording aCSF containing (in mM): 124 NaCl, 2.5 KCl, 2 CaCl_2_, 1.2 NaH_2_PO_4_, 24 NaHCO_3_, 2 MgSO_4_, 12.5 D-glucose, 5 HEPES (pH 7.3). Recording aCSF was passed through an in-line heater at a flow rate of 1.5–2.5 ml/min, and the temperature was held constant at 30°C.

### 2.7. Whole-cell patch-clamp recording

Whole-cell responses were recorded as described previously (Chen et al., 2021). Briefly, whole-cell patch-clamp recordings were made from MSNs in the DLS. MSNs were morphologically and electrophysiologically identified. To assess the intrinsic membrane and AP properties of MSNs, glass pipettes (5–6 MΩ resistance) filled with internal solution containing (in mM) 110 K-gluconic acid, 10 NaCl, 1 MgCl_2_, 10 EGTA, 40 HEPES, 2 Mg-ATP, 0.3 Na-GTP (pH 7.3; 280–300 mOsm) were used; in some cases, 0.1% biocytin (B4261, Sigma, USA) was added to the intracellular solution to visualize patched cells. The voltage responses were measured at steady state; a series of 20-or 1,000-ms depolarizing currents (from +40 pA to +440 pA with increments of 40 pA) was applied for the remaining measurements. Single AP properties, such as AP threshold, amplitude, half-width, rise and decay time, and AHP amplitude, were measured for the second evoked AP in a 20-ms current-clamp series. Finally, a series of 1,000-ms hyperpolarizing and depolarizing steps was used to assess repetitive AP firing. For recording of miniature excitatory postsynaptic currents (mEPSCs) and evoked excitatory postsynaptic currents (eEPSCs), the recording aCSF was supplemented with 1 μM tetrodotoxin (to block sodium current) and 50 μM picrotoxin (P-1675, Sigma, USA; to block GABA_A_ receptors). The synaptic response was recorded with glass pipettes (5–6 MΩ resistance) filled with internal solution containing the following (in mM): 100 CsMeSO_4_, 10 NaCl, 10 TEA-Cl, 1 MgCl_2_, 10 EGTA, 40 HEPES, 2 Mg-ATP, 0.3 Na_2_-GTP, 3 QX-314 (pH 7.3; 280–300 mOsm). To assess eEPSCs, electrical field stimulation was achieved using a bipolar stimulation electrode placed in the dorsolateral corpus callosum to evoke glutamate release from the corticostriatal pathway. The MSNs to be recorded were located 300 ~ 400 μm away from the stimulation site. To construct input–output (I-O) curves for AMPAR-mediated eEPSCs, electrical stimulation current intensity was varied from 0.05 to 0.4 mA in 0.05 mA steps for each neuron. Paired pulses of AMPAR-mediated eEPSCs were delivered at 50 ms inter-pulse intervals. To construct stimulation-response curves for N-methyl-D-aspartate receptor (NMDAR)-mediated eEPSCs, electrical stimulation current intensity was varied from 0.1 to 0.6 mA in 0.1 mA steps for each neuron. To isolate AMPAR-and NMDAR-mediated eEPSCs, MSNs were voltage-clamped at −70 mV and + 40 mV in the presence of 50 μM picrotoxin. The AMPAR-mediated eEPSC amplitude was measured as the peak eEPSC amplitude at −70 mV, while NMDAR-mediated eEPSCs were estimated as the eEPSC amplitude 50 ms after the stimulation artifact at +40 mV. eEPSC paired-pulse ratios (PPRs) were measured by dividing the EPSC amplitude evoked by the second stimulus by the EPSC amplitude evoked by the first stimulus (*R*_2_/*R*_1_). For (RS)-3, 5-dihydroxyphenylglycine (DHPG)-induced LTD whole-cell experiments, MSNs were voltage-clamped at −70 mV in 95% O_2_/5% CO_2_-saturated recording aCSF with 50 μM picrotoxin. After recording 15–20 min of stable baseline, DHPG-induced LTD was induced by bath application of 100 μM DHPG for 10 min. The recording was continued for a further 30 min to monitor the induction and maintenance of LTD. All data were acquired with a Digidata 1440A interface and pClamp 10.4 software (Molecular Devices, USA) and a MultiClamp 700B amplifier (Molecular Devices, USA). Data were analyzed using Clampfit 10.4 (Molecular Devices, USA), Mini Analysis program (Synaptosoft, USA) and GraphPad Prism 8.0.2 (GraphPad, USA).

### 2.8. Field potential recording

The procedures for field potential recording were described previously (Nazzaro et al., 2012; Misrani et al., 2019). Briefly, a single slice was transferred to a chamber perfused with 95% O_2_/5% CO_2_-saturated recording aCSF without 50 μM picrotoxin. The field potential recording glass pipette (3–4 MΩ resistance) filled with aCSF was placed in the DLS and a bipolar electrode was placed in the corpus callosum. After recording 15–20 min of stable baseline, LTD was induced by four trains of HFS (100 Hz, 1 s) with an inter-train interval of 10 s, and recording was continued for a further 60 min to monitor the induction and maintenance of LTD in the corticostriatal pathway. All data acquisition was done using a Digidata 1,550 interface and pClampex 10.4 software. Data were analyzed using Clampfit 10.4, SPSS software and GraphPad Prism 8.0.2.

### 2.9. Biocytin labeling, image collection and analysis

Patched MSNs were visualized using biocytin for morphological analyses as described (Jiang et al., 2022). After whole-cell voltage-clamp recording, slices containing biocytin-filled MSNs were transferred to 4% paraformaldehyde (PFA) and fixed overnight at 4°C. Slices were washed (three times, 5 min each) with PBS and permeabilized in 1% Triton-X100/PBS overnight at 4°C. Subsequently, sections were incubated at 4°C overnight in Streptavidin Alexa 488 (S11223, Invitrogen, USA) diluted 1:1000 in 0.3% Triton-X100/PBS, then given three 5-min washes with PBS and mounted on glass slides. Images were collected with a confocal microscope (LSM-800, Zeiss, Germany) using 20× (2 μm Z-step) and 40× oil immersion (0.2 μm Z-step) objectives at 1024 × 1,024 pixels by sequential scanning and then processed using ZEN (Zeiss, Germany) and ImageJ software (NIH, USA). For the morphological analysis, we selected 9–11 animals per experimental group (blind to genotype), 2–3 MSNs per animal and 4–5 individual secondary dendrites (at least 50 μm from the soma; >20 μm long) per cell of comparable diameter. Dendrite arborization and spine density were quantified by an independent investigator. Dendritic spines were classified based on the spine length and spine head width, as follows: mushroom (with spine head, head width/length ratio > 0.5); thin (with spine head, length > 1.2 μm, head width/length ratio < 0.5); stubby (length < 1.2 μm, with spine head, head width/length ratio < 0.5; without head, not applicable); filopodia (without spine head, length > 1.2 μm) (Llano et al., 2015; Yasuda et al., 2020). In addition to the four classical spine subtypes, we define varicosity as dendritic segments displaying varicose swellings (dendritic diameter > 2 μm) with spine loss (Westrum et al., 1964; Isokawa, 1997; Swann et al., 2000; Maiti et al., 2015). Dendritic spine density was calculated as the number of spines per unit length.

### 2.10. Immunofluorescent staining of striatal slices

Brain slices were prepared as for electrophysiological recordings. Slices were then transferred to a treatment chamber containing recording aCSF and treated with 100 μM DHPG or aCSF alone for 10 min at RT. For immunofluorescence, slices were allowed to recover for 10 min in recording aCSF and then fixed in 4% PFA/0.2% glutaraldehyde in PBS for 24 h, after which they were transferred to 30% sucrose in PBS until saturated. The slices were embedded in Tissue-Tek O.C.T Compound (4,583; SAKURA, USA) and stored at-80°C before being sliced into 20 μm coronal cryostat sections at-18°C (CM3050, Lecia, Germany).

The procedures for immunohistochemistry were described previously (Zhou et al., 2018; Chen et al., 2021). Briefly, mice were anesthetized with 10% chloral hydrate and subjected to cardiac perfusion with 95% O_2_/5% CO_2_-saturated recording aCSF followed by 4% PFA/0.2% glutaraldehyde in PBS. Sections were washed three times with PBS, permeabilized with 0.05% Triton X-100 in PBS for 2 h, blocked with 10% donkey serum, incubated with primary antibodies overnight at 4°C and then incubated with appropriate secondary antibodies and DAPI at RT for 2 h. Sections were subsequently washed and mounted on glass slides. For confocal microscopy, images were acquired using a Zeiss LSM-800 fitted with a 40 × oil immersion (2 μm Z-step) objective at a pixel resolution of 1,024 × 1,024. The image acquisition settings were the same for all scans when fluorescence intensity was compared. Images were analyzed using ZEN (Zeiss) and ImageJ software (NIH).

### 2.11. Statistics and reproducibility

All data are expressed as the mean ± standard error of the mean (SEM) and are statistically evaluated by Student’s *t*-test, ANOVA (one-way, two-way, two-way repeated measures) with Tukey’s or Sidak’s post-hoc multiple comparison test or two-sample Kolmogorov–Smirnov test using GraphPad Prism and SPSS. *p* < 0.05 was considered significant (*p < 0.05, **p < 0.01, ***p < 0.001). Mean ± SEM values, sample size, *p*-values and statistical methods are defined in the respective results and figure legends.

## 3. Results

### 3.1. CYLD affects the morphology and physiological features of MSNs

**Figure 1:**
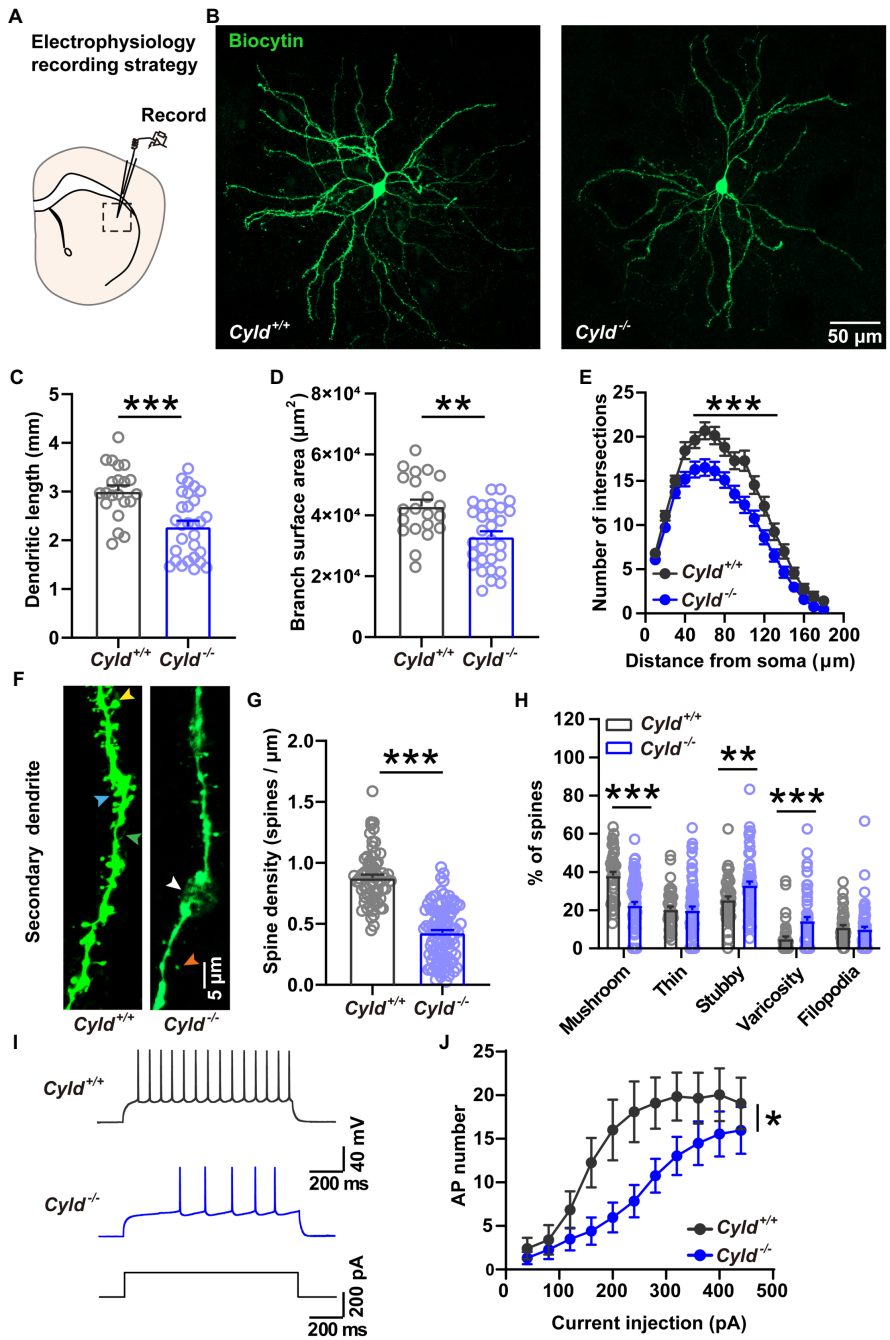
CYLD modulates the morphology and firing activity of MSNs in the DLS. **(A)** Diagram of the electrophysiological recording strategy in acute striatal slices. **(B)** Representative images of individual MSNs from *Cyld^+/+^* or *Cyld^−/−^* mice, filled with biocytin *via* a patch pipette. Neurons were imaged with a confocal microscope system and images were used to analyze dendritic and spine morphology. Magnification: 20 ×, scale = 50 μm. **(C)** Bar graphs showing that the dendritic length of MSNs decreases in *Cyld^−/−^* mice. **(D)** Bar graphs showing that the arborization surface area of MSNs decreases in *Cyld^−/−^* mice. **(E)** Sholl analysis demonstrating that reduced arborization is statistically significant in the region 40–120 μm from the cell soma in *Cyld^−/−^* MSNs. Points represent individual neurons (*n* = 21 neurons from 11 *Cyld^+/+^* mice, *n* = 29 neurons from 6 *Cyld^−/−^* mice) in **(B–D)**. **(F)** Representative confocal images of secondary dendritic spines filled with biocytin in *Cyld^+/+^* or *Cyld^−/−^* MSNs. Dendritic spines were classified into five different types: mushroom (yellow arrowhead), thin (orange arrowhead), stubby (blue arrowhead), varicosity (white arrowhead) and filopodia (green arrowhead). Abnormal varicose dendrites absorbing spines were observed in *Cyld^−/−^* MSNs. Magnification: 40 x, scale = 5 μm. **(G)** Bar graphs showing that the dendritic spine density of MSNs decreases in *Cyld^−/−^* mice. Spine density of individual secondary dendrites in each neuron was calculated as the number of spines per unit length, with each point representing a single dendrite (*n* = 66 dendrites from *Cyld^+/+^*, *n* = 100 dendrites from *Cyld^−/−^*). **(H)** Bar graphs showing more stubby spines and varicosity, but fewer mushroom spines and filopodia, in *Cyld^−/−^* MSNs than in *Cyld^+/+^* MSNs. **(I)** Using a current-clamp configuration, the membrane voltage response to 1,000-ms injections in +200 pA current steps in representative MSNs from *Cyld^+/+^* and *Cyld^−/−^* mice is shown. **(J)** Responses to depolarizing current steps (+40 to +400 pA, 40 pA steps, 1,000 ms duration) lead to a decrease in AP frequency in *Cyld^−/−^* MSNs (*n* = 21 neurons from 7 *Cyld^+/+^* mice, *n* = 23 neurons from 6 *Cyld^−/−^* mice). Data are presented as the mean ± SEM; *p < 0.05, **p < 0.01, ***p < 0.001.

CYLD is highly expressed in the dorsal striatum and regulates the morphogenesis of dendrites and spines. To determine whether CYLD plays a role in neurite complexity and spine morphology of MSNs in the DLS, we examined MSN morphology by filling the neurons with biocytin during whole-cell patch-clamp recordings. We measured the complexity of neuronal dendritic arborization in MSNs using three parameters: the length of dendrites, the surface area of dendrite coverage and the number of dendrite intersections at various distances from the cell body (Sholl’s analysis) (Ferreira et al., 2014; Wang et al., 2019). We found that CYLD deficiency induced a significant reduction in dendrite length (Figures 1A–C, *Cyld^+/+^*: 3.005 ± 0.120 mm, *Cyld^−/−^*: 2.282 ± 0.120 mm, *t* = 4.163, *p* = 0.0001, unpaired Student’s *t*-test) and dendrite surface area (Figure 1D, *Cyld^+/+^*: 4.298 × 10^4^ ± 2.203 × 10^3^, *Cyld^−/−^*: 3.294 × 10^4^ ± 1.851 × 10^3^, *t* = 3.497, *p* = 0.001, unpaired Student’s *t*-test), as well as the number of branches (Figure 1E, interaction between genotype and distance, *F*_(17, 814)_ = 2.312, *p* = 0.002; main effect of genotype, *F*_(1, 48)_ = 14.450, *p* = 0.0004; main effect of distance, *F*_(3.202, 153.3)_ = 202.200, *p* = 0.188, two-way repeated measures ANOVA).

To determine whether the dendritic spines, minute protrusions on dendrites, were altered, we segregated dendritic protrusions into different groups: mushroom spine, thin spine, stubby spine, varicosity and filopodia, based on the spine length, head width and dendrite diameter (see Section 2) (Swann et al., 2000; Maiti et al., 2015; Bello-Medina et al., 2016). We found abnormal spine loss with dendritic beading (Figures 1F,G, *Cyld^+/+^*: 0.877 ± 0.027, *Cyld^−/−^*: 0.427 ± 0.023, *t* = 12.640, *p* < 0.0001, unpaired Student’s *t*-test), as well as spine paramorphia in *Cyld^−/−^* MSNs. The mature mushroom spine ratio was also significantly decreased (Figure 1H, *Cyld^+/+^*: 38.096 ± 2.010, *Cyld^−/−^*: 22.518 ± 1.756, *t* = 5.746, *p* < 0.0001, unpaired Student’s *t*-test). In contrast, the proportion of immature stubby spine and pathological varicosity, was significantly increased in *Cyld^−/−^* MSNs (Figure 1H, stubby spine, *Cyld^+/+^*: 25.451 ± 1.733, *Cyld^−/−^*: 33.132 ± 1.908, *t* = 2.801, *p* = 0.006, unpaired Student’s *t*-test; varicosity, *Cyld^+/+^*: 4.999 ± 1.2056, *Cyld^−/−^*: 14.386 ± 2.060, *t* = 3.443, *p* = 0.0008, unpaired Student’s *t*-test). These data indicate that stable synaptic contacts were decreased in *Cyld^−/−^* MSNs.

APs are the fundamental electrical signals used by the central nervous system to relay information (Bean, 2007). Therefore, we measured APs in MSNs to investigate how the above structural alterations are associated with functional modification. First, we analyzed the intrinsic electrical properties of a single AP. The AP threshold, half-width and rise time, as well as the amplitude of the after-hyperpolarization potential (AHP), were significantly increased in *Cyld^−/−^* MSNs, suggesting that excitability is subnormal in CYLD knockout MSNs (Table 2). Furthermore, we measured intrinsic tonic firing activity in MSNs by assessing their response to depolarizing current injections (ranging from +40 to +440 pA, 40 pA steps, 1,000 ms) and found that AP firing was decreased in *Cyld^−/−^* MSNs (Figures 1I,J, interaction between genotype and current injection, *F*_(10, 430)_ = 1.607, *p* = 0.102; main effect of genotype, *F*_(1, 43)_ = 6.784, *p* = 0.013; main effect of current injection, *F*_(2.004, 86.17)_ = 21.500, *p* < 0.0001, two-way repeated measures ANOVA). These data suggest that CYLD has a role in MSN intrinsic tonic firing activity under basal conditions.

**Table 2:** Intrinsic properties of MSNs in DLS.

Parameters	*Cyld*^+/+^ (*n* = 21 neurons from 7 mice)	*Cyld*^−/−^ (*n* = 24 neurons from 6 mice)
RMP (mV)	−66.726 ± 1.976	−66.430 ± 1.867
Rheobase (nA)	154.000 ± 18.158	189.565 ± 22.301
AP threshold (mV)	−39.127 ± 1.752	−34.180 ± 1.199*
AP amplitude (mV)	67.566 ± 3.490	68.283 ± 2.369
Half-width (ms)	1.540 ± 0.109	1.798 ± 0.067*
Rise time (ms)	0.501 ± 0.032	0.611 ± 0.026**
Decay time (ms)	1.533 ± 0.032	1.654 ± 0.111
AHP amplitude (mV)	−7.317 ± 0.601	−9.346 ± 0.479*

RMP, resting membrane potential; AP, action potential; AHP, after-hyperpolarization potential; *n* is number of neurons. Values are mean ± SEM, *p < 0.05; **p < 0.01; unpaired Student’s *t*-test.

### 3.2. CYLD regulates AMPAR-mediated excitatory synaptic transmission

**Figure 2:**
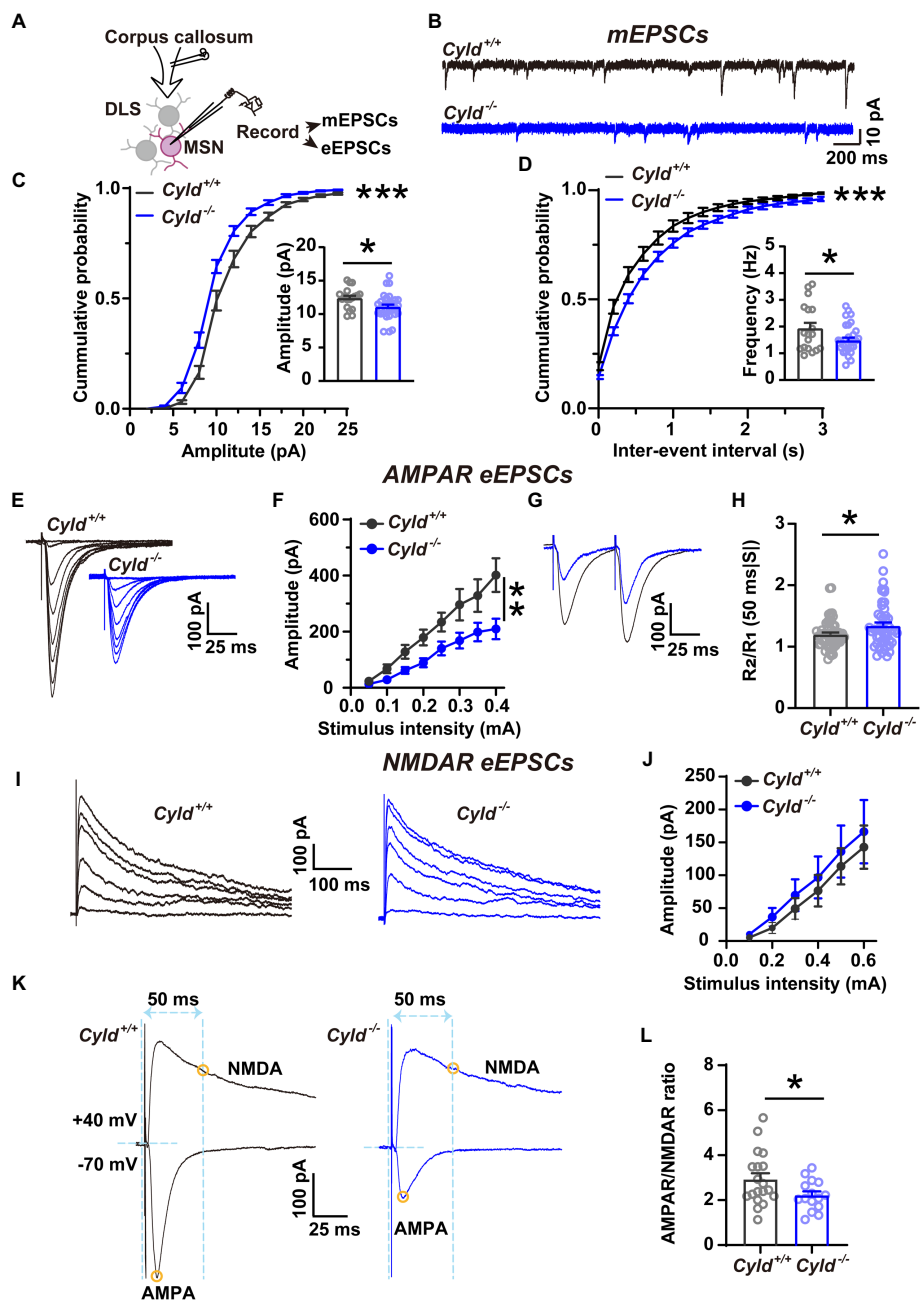
Corticostriatal excitatory synaptic transmission is decreased in *Cyld^−/−^* MSNs. **(A)** Diagram of the electrophysiological recording strategy in acute striatal slices. **(B)** Whole-cell voltage-clamp recording traces of mEPSCs from MSNs in *Cyld^+/+^* (black) or *Cyld^−/−^* (blue) mice. **(C,D)** Cumulative distribution and quantification of the average mEPSC amplitude **(C)** and frequency **(D)** in *Cyld^−/−^* MSNs, showing significantly decreased mEPSC amplitude and frequency in *Cyld^−/−^* MSNs (*n* = 18 neurons from 8 *Cyld^+/+^* mice, *n* = 28 neurons from 9 *Cyld^−/−^* mice). **(E)** Typical examples of AMPAR-mediated eEPSCs recorded from MSNs after stimulation of the dorsolateral corpus callosum in *Cyld^+/+^* and *Cyld^−/−^*. **(F)** I-O curves for AMPAR-mediated eEPSCs at −70 mV were obtained by applying stimuli from 0.05 to 0.4 mA (*n* = 43 neurons from 22 *Cyld^+/+^* mice, *n* = 50 neurons from 17 *Cyld^−/−^* mice). **(G)** Representative traces of AMPAR-mediated eEPSCs at 50-ms inter-stimulus intervals from MSNs in *Cyld^+/+^* and *Cyld^−/−^* mice. **(H)** Bar-graphs summarizing the PPRs of AMPAR-mediated eEPSCs at 50-ms inter-stimulus intervals (*n* = 57 neurons from 22 *Cyld^+/+^* mice, *n* = 51 neurons from 17 *Cyld^−/−^* mice). **(I)** Typical examples of NMDAR-mediated eEPSCs recorded from MSNs after stimulating the dorsolateral corpus callosum in *Cyld^+/+^* and *Cyld^−/−^* mice. **(J)** I-O curves for NMDAR-mediated eEPSCs at +40 mV obtained by applying stimuli from 0.05 to 0.6 mA (*n* = 9 neurons from 5 *Cyld^+/+^* mice, *n* = 14 neurons from 5 *Cyld^−/−^* mice). **(K)** Representative traces of eEPSCs in the same MSNs. Yellow circles indicate that AMPAR-mediated eEPSCs were measured at the peak at a holding potential of −70 mV, while NMDAR-eEPSCs were measured 50 ms after stimulation at a holding potential of +40 mV. **(L)** The ratio of AMPAR-to NMDAR-mediated eEPSCs is reduced in *Cyld^−/−^* MSNs (*n* = 13 neurons from 8 *Cyld^+/+^* mice, *n* = 15 neurons from 8 *Cyld^−/−^* mice). Data are presented as mean ± SEM; *p < 0.05, **p < 0.01, ***p < 0.001.

We further asked whether the function of CYLD is also critical for excitatory synaptic connective function in DLS. To address this question, we carried out whole-cell patch-clamp experiments to record mEPSCs and eEPSCs (Figure 2A). We found that the amplitude and frequency of mEPSCs in MSNs were significantly decreased in *Cyld^−/−^* mice, indicating that there were fewer functional synapses in *Cyld^−/−^* MSNs, contributing to a decreased glutamate receptor and synaptic drive onto MSNs (Figures 2B–D, cumulative frequency distributions of amplitude, D = 0.19, *p* < 0.0001, two-sample Kolmogorov–Smirnov test; amplitude, *Cyld^+/+^*: 12.370 ± 0.375, *Cyld^−/−^*: 11.060 ± 0.370, *t* = 2.382, *p* = 0.022; cumulative frequency distributions of frequency, D = 0.241, *p* < 0.0001, two-sample Kolmogorov–Smirnov test; frequency, *Cyld^+/+^*: 1.931 ± 0.210, *Cyld^−/−^*: 1.477 ± 0.110, *t* = 2.091, *p* = 0.042, unpaired Student’s *t*-test).

AMPARs and NMDARs are two major types of glutamate receptors involved in excitatory synaptic transmission. AMPARs mediate the postsynaptic depolarization that initiates neuronal firing and also mediate the majority of fast synaptic transmission, whereas NMDARs mediate a slow component of excitatory potentials at glutamatergic synapses (Bredt and Nicoll, 2003; Popescu and Auerbach, 2003). As a further test for the effects of CYLD deficiency on MSN basal AMPAR-or NMDAR-mediated synaptic properties, we examined the I-O curves with incremental stimulus intensities: a significant decrease in the amplitude of AMPAR-mediated eEPSCs at −70 mV in *Cyld^−/−^* MSNs was observed (Figures 2E,F, interaction between genotype and stimulation, *F*_(7, 595)_ = 4.640, *p* < 0.0001, main effect of genotype, *F*_(1, 91)_ = 7.936, *p* = 0.006; main effect of stimulation, *F*_(7, 595)_ = 61.710, *p* < 0.0001, two-way repeated measures ANOVA), while NMDAR-mediated eEPCSs at +40 mV showed no difference between the genotypes (Figures 2I,J, interaction between genotype and stimulation, *F*_(5, 104)_ = 0.077, *p* = 0.996, main effect of genotype, *F*_(1, 21)_ = 0.237, *p* = 0.631; main effect of stimulation, *F*_(5, 104)_ = 23.460, *p* < 0.0001, two-way repeated measures ANOVA). Therefore, the AMPAR/NMDAR ratio was significantly reduced in *Cyld^−/−^* mice (Figures 2K,L, *Cyld^+/+^*: 3.101 ± 0.380, *Cyld^−/−^*: 2.216 ± 0.173, *t* = 2.222, *p* = 0.035, unpaired Student’s *t*-test) as a result of the decrease in AMPAR-mediated excitatory synaptic transmission.

Given that both the probability of presynaptic glutamate release and the number of functional synapses contribute to the frequency of mEPSCs, we measured PPRs, determined by the amplitude of the response to the second stimulus divided by the amplitude of the first one (*R*_2_/*R*_1_). This reflects neurotransmitter release probability, with a low PPR signifying a high initial release probability (Dobrunz and Stevens, 1997; Zinebi et al., 2001). The PPR of AMPAR-mediated eEPSCs at 50-ms inter-stimulus intervals was significantly increased in *Cyld^−/−^* mice, suggesting a reduced probability of evoked presynaptic glutamate release from cortical terminals (Figures 2G,H, *Cyld^+/+^*: 1.200 ± 0.030, *Cyld^−/−^*: 1.342 ± 0.052, *t* = 2.426, *p* = 0.017, unpaired Student’s *t*-test). Thus far, the data suggest that CYLD deficiency alters both morphology and synaptic activity of MSNs.

### 3.3. CYLD deficiency reduces surface levels of GluA1 and GluA2

**Figure 3:**
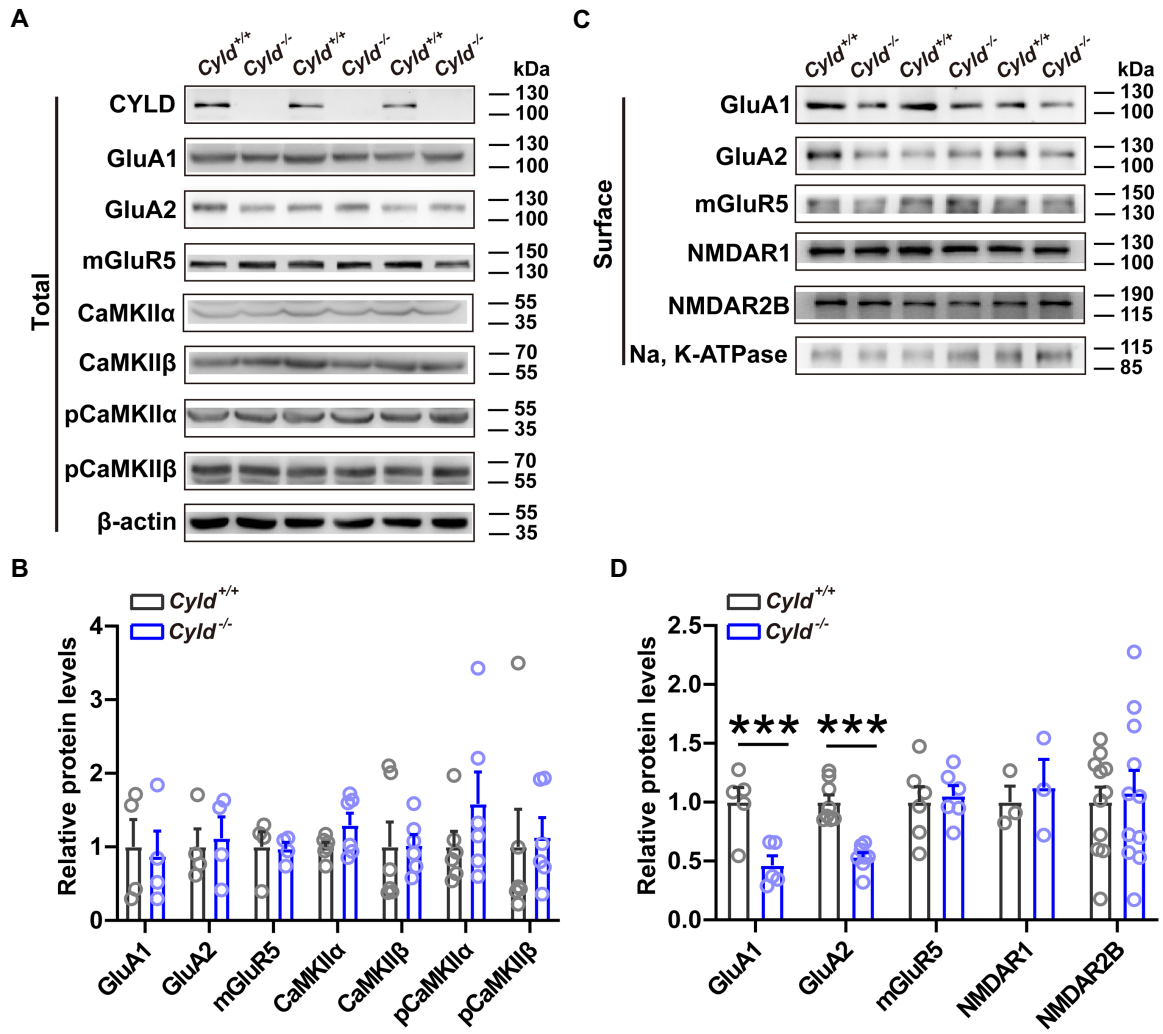
CYLD stabilizes AMPARs. **(A)** Representative immunoblots of total protein isolated from the DLS of *Cyld^+/+^* and *Cyld^−/−^* littermates. **(B)** Quantification of immunoblots in **(A)** reveals that total protein expression levels remain unchanged between genotypes (GluA1, GluA2 and mGluR5: *n* = 4 per group; CaMKIIα, CaMKIIβ, pCaMKIIα and pCaMKIIβ: *n* = 6 per group). **(C)** Representative immunoblots of surface protein isolated from the DLS of *Cyld^+/+^* and *Cyld^−/−^* littermates. **(D)** Quantification of immunoblots in **(C)** reveals a significant decrease in surface GluA1 and GluA2 protein levels in *Cyld^−/−^* mice (GluA1: *n* = 5 per group; GluA2: *n* = 8 per group; NMDAR1 and mGluR5: *n* = 3 per group; NMDAR2B: *n* = 11 per group). β-actin and Na, K-ATPase were used as loading controls. Data are presented as the mean ± SEM; ***p < 0.001.

To investigate whether there were any molecular alterations at synapses that parallel the electrophysiological and morphological impairments noted above, we evaluated total and plasma membrane excitatory synaptic protein levels in DLS using western blotting. We found that although the total amount of GluA1 and GluA2 expressed did not change significantly between genotypes (Figures 3A,B, GluA1, *Cyld^+/+^*: 1.000 ± 0.372, *Cyld^−/−^*: 0.8749 ± 0.3416, *t* = 0.248, *p* = 0.8126; GluA2, *Cyld^+/+^*: 1.000 ± 0.244, *Cyld^−/−^*: 1.120 ± 0.287, *t* = 0.318, *p* = 0.761, unpaired Student’s *t*-test), levels of surface-expressed GluA1 and GluA2 were significantly reduced in *Cyld^−/−^* mice (Figures 3C,D, GluA1, *Cyld^+/+^*: 1.000 ± 0.123, *Cyld^−/−^*: 0.4646 ± 0.08106, *t* = 3.635, *p* = 0.0066; GluA2, *Cyld^+/+^*: 1.000 ± 0.0618, *Cyld^−/−^*: 0.535 ± 0.037, *t* = 6.455, *p* < 0.0001, unpaired Student’s *t-*test), suggesting a role for CYLD in AMPAR trafficking. We also measured levels of total calcium/calmodulin-dependent protein kinase II (CaMKII), pCaMKII (phosphor T286), which regulates the delivery and removal of AMPARs (Opazo et al., 2010; Hoerndli et al., 2015). However, there were no significant differences between genotypes (Figures 3A,B, CaMKIIα, *Cyld^+/+^*: 1.000 ± 0.061, *Cyld^−/−^*: 1.296 ± 0.162, *t* = 1.715, *p* = 0.117; CaMKIIβ, *Cyld^+/+^*: 1.000 ± 0.337, *Cyld^−/−^*: 1.022 ± 0.148, *t* = 0.059, *p* = 0.954; pCaMKIIα, *Cyld^+/+^*: 1.000 ± 0.211, *Cyld^−/−^*: 1.586 ± 0.433, *t* = 1.218, *p* = 0.251; pCaMKIIβ, *Cyld^+/+^*: 1.000 ± 0.511, *Cyld^−/−^*: 1.130 ± 0.268, *t* = 0.226, *p* = 0.826). Given that mGluRs, especially mGluR5, are highly enriched in the striatum and mediate long-term synaptic plasticity (Moussawi et al., 2009; Knackstedt et al., 2014), we tested levels of total and surface mGluR5, but found no significant differences between genotypes (Figures 3A–D, total amount of mGluR5, *Cyld^+/+^*: 1.000 ± 0.205, *Cyld^−/−^*: 0.973 ± 0.088, *t* = 0.119, *p* = 0. 909; surface-expressed mGluR5, *Cyld^+/+^*: 1.000 ± 0.114, *Cyld^−/−^*: 1.053 ± 0.076, *t* = 0.337, *p* = 0. 743, unpaired Student’s *t*-test). Consistent with the unchanged amplitude of NMDAR-mediated eEPSCs noted above, we observed unchanged levels of surface-expressed NMDAR1 and NMDAR2B, key subunits of NMDARs (Figures 3C,D, NMDAR1, *Cyld^+/+^*: 1.000 ± 0.136, *Cyld^−/−^*: 1.124 ± 0.239, *t* = 0.452, *p* = 0. 675; NMDAR2B, *Cyld^+/+^*: 1.000 ± 0.128, *Cyld^−/−^*: 1.079 ± 0.191, *t* = 0.344, *p* = 0.734, unpaired Student’s *t*-test).

### 3.4. CYLD regulates K63-linked ubiquitination of GluA1 and GluA2

**Figure 4:**
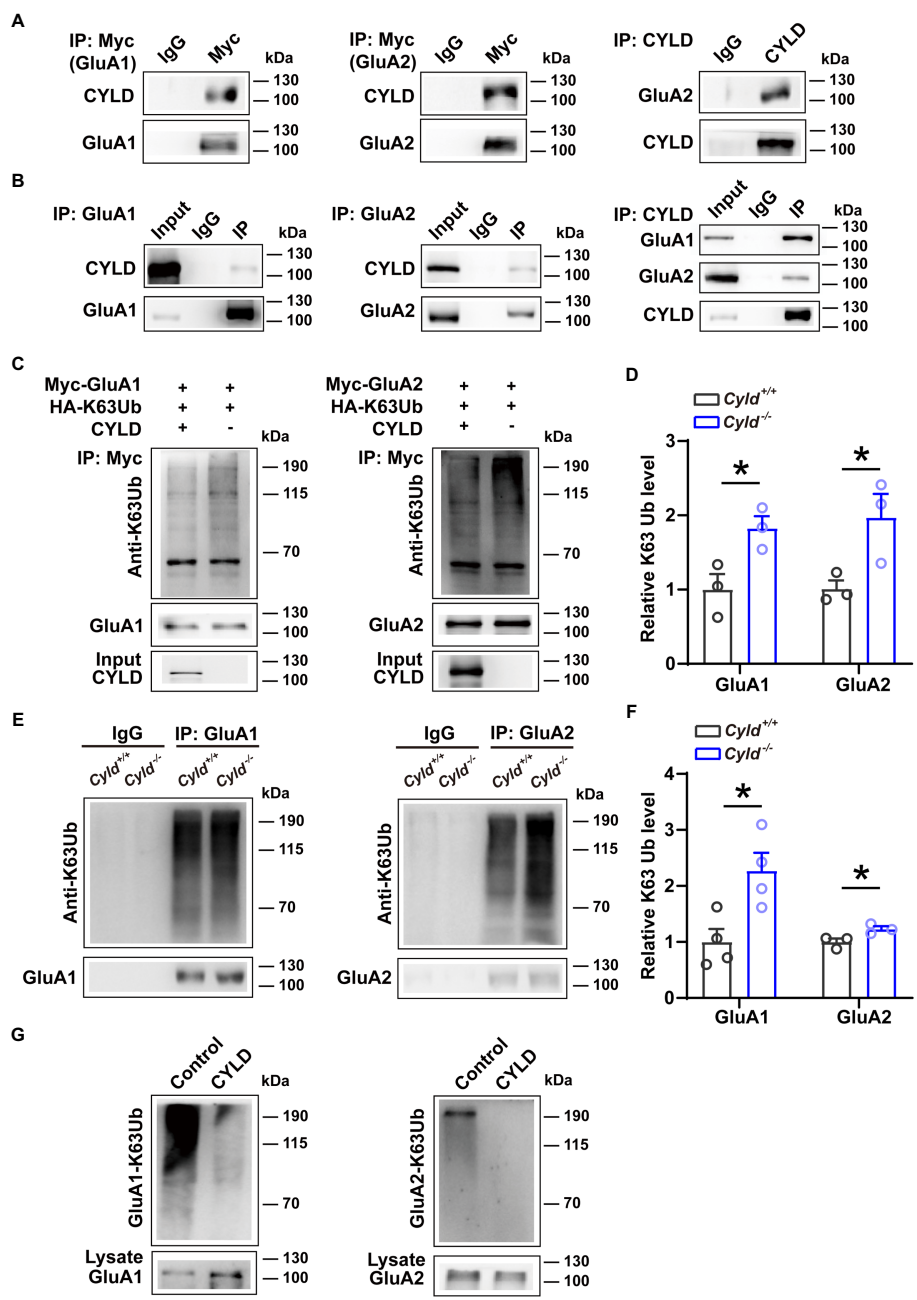
CYLD regulates GluA1 and GluA2 K63-ubiquitination. **(A)** HEK293 cells were cotransfected with CYLD and Myc-GluA1 or Myc-GluA2 expression vectors. The cell lysates were immunoprecipitated using anti-Myc and anti-CYLD antibodies and immunoblotted with anti-CYLD and anti-GluA2 antibodies. **(B)** Interaction between endogenous CYLD and GluA1 and GluA2 in the mouse brain, analyzed by immunoprecipitation. Rabbit IgG was used as a negative control in immunoprecipitation experiments. **(C)** HEK293 cells were cotransfected with expression vectors for various combinations of Myc-GluA1 (left) or Myc-GluA2 (right), HA-K63Ub and CYLD, followed by immunoprecipitation with anti-Myc and immunoblotting with anti-K63Ub. **(D)** Quantification of immunoblots in **(C)** shows a reduction in K63Ub conjugated to GluA1 (left) and GluA2 (right) by CYLD in HEK293 cells. **(E)** DLS lysates from *Cyld^+/+^* and *Cyld^−/−^* littermates were immunoprecipitated with anti-GluA1 (left) or anti-GluA2 (right) antibodies and immunoblotted with anti-K63Ub. Rabbit IgG was used as a negative control in immunoprecipitation experiments. **(F)** Quantification of immunoblots in **(E)** reveals increased GluA1 and GluA2 K63 ubiquitination in *Cyld^−/−^* mice (*n* = 4 *Cyld^+/+^* mice, *n* = 4 *Cyld^−/−^* mice). **(G)** *In vitro* deubiquitination assays showing that CYLD removes GluA1 (left) and GluA2 (right) K63Ub chains. Data are presented as the mean ± SEM; *p<0.05.

To understand the molecular mechanism mediating levels of surface-expressed GluA1 and GluA2 in *Cyld^−/−^* mice, we investigated the potential physical and functional associations of GluA1, GluA2 and CYLD. We first assessed the interaction of exogenously expressed GluA1 and GluA2 with CYLD in HEK293 cells. We co-transfected HEK293 cells with CYLD and Myc-GluA1 or Myc-GluA2. Cell lysates were immunoprecipitated using either anti-Myc or anti-CYLD antibodies, and immunoblotted with anti-CYLD and anti-GluA2. The results show that CYLD interacts with GluA1 and GluA2 (Figure 4A). To test whether these interactions occur endogenously, we performed co-immunoprecipitation (co-IP) experiment with CYLD using brain tissue lysates and obtained co-precipitation of both GluA1 and GluA2. Both GluA1 and GluA2 efficiently co-precipitated CYLD, suggesting interaction between endogenous CYLD and GluA1 or GluA2 in the converse IP experiment (Figure 4B).

We further evaluated the functional relevance of GluA1 and GluA2 interacting with CYLD. Given that K63Ub chains are the primary posttranslational modification of GluA1 and GluA2 (Widagdo et al., 2017), and that CYLD specifically cleaves K63Ub chains (Sato et al., 2015), we tested whether CYLD deficiency affects the levels of GluA1 and GluA2 K63-linked ubiquitination. HEK293 cells were co-transfected with Myc-GluA1 or Myc-GluA2 and HA-K63Ub, with or without CYLD. We precipitated GluA1 and GluA2 using anti-Myc antibody and immunoblotted with anti-K63Ub. For both GluA1 and GluA2, K63-polyubiquitination was diminished in the presence of CYLD, suggesting that CYLD reduced K63Ub conjugation to GluA1 and GluA2 in HEK293 cells (Figures 4C,D, GluA1-K63Ub, *Cyld^+/+^*: 1.000 ± 0.179, *Cyld^−/−^*: 1.826 ± 0.140, *t* = 3.145, *p* = 0.035; GluA2-K63Ub, *Cyld^+/+^*: 1.000 ± 0.097, *Cyld^−/−^*: 1.972 ± 0.276, *t* = 2.848, *p* = 0.0467, unpaired Student’s *t*-test). To further test whether K63-linked ubiquitination of GluA1 or GluA2 was defective in *Cyld^−/−^* mice, we separately immunoprecipitated GluA1 and GluA2 using anti-GluA1 and anti-GluA2 antibody and immunoblotted with anti-K63Ub from *Cyld^+/+^* and *Cyld^−/−^* mice. As expected, we observed that CYLD knockout increased K63-linked ubiquitination of GluA1 and GluA2 (Figures 4E,F, GluA1-K63Ub, *Cyld^+/+^*: 1.000 ± 0.230, *Cyld^−/−^*: 2.272 ± 0.320, *t* = 3.226, *p* = 0.018; GluA2-K63Ub, *Cyld^+/+^*: 1.000 ± 0.062, *Cyld^−/−^*: 1.231 ± 0.049, *t* = 2.922, *p* = 0.043, unpaired Student’s *t*-test). Using *in vitro* deubiquination assays, we co-expressed Myc-GluA1 or Myc-GluA2 with HA-K63Ub. GluA1 and GluA2 were purified by IP using anti-Myc antibody, eluted with Myc peptide, then incubated with or without CYLD. As previously, we observed that CYLD removed GluA1 and GluA2 K63Ub chains (Figure 4G).

### 3.5. CYLD deficiency reduces DHPG-triggered removal of GluA1

**Figure 5:**
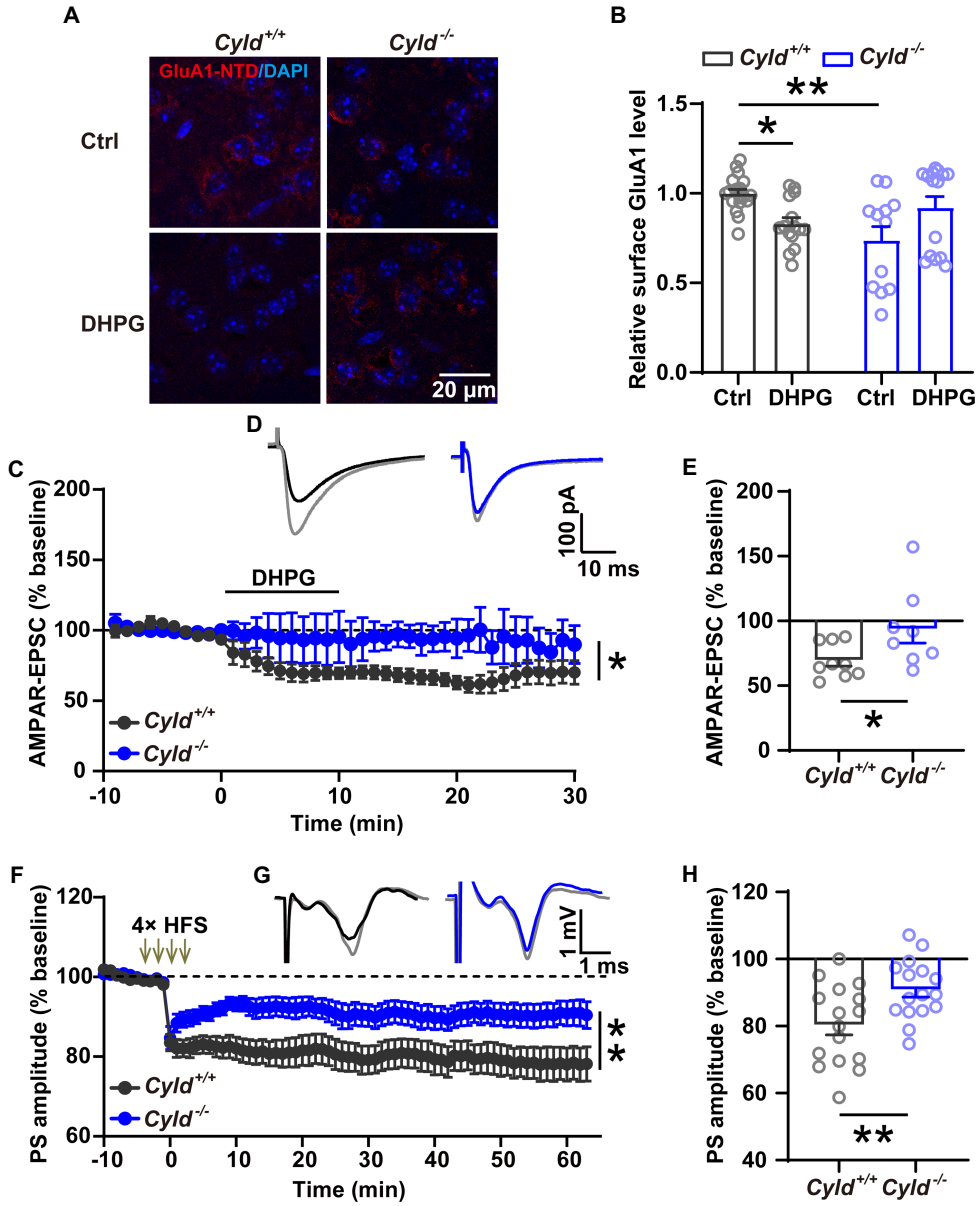
CYLD deficiency impairs both DHPG-and HFS-induced LTD in the DLS. **(A)** Representative confocal images showing surface GluA1 (red) and DAPI (blue) in acute striatal slices treated with or without DHPG (Ctrl) in *Cyld^+/+^* and *Cyld^−/−^* mice. Scale bar: 20 μm. **(B)** Normalized surface GluA1 in DHPG-treated slices compared to Ctrl (*n* = 3 *Cyld^+/+^* mice, *n* = 3 *Cyld^−/−^* mice). **(C)** Absence of DHPG-induced LTD in whole-cell recordings in *Cyld^−/−^* mice. **(D)** Representative traces of AMPAR-mediated EPSCs evoked in a MSN showing the baseline (gray) and 1–30 min after addition of DHPG in *Cyld^+/+^* (black) and *Cyld^−/−^* (blue) mice. **(E)** The graph shows the mean AMPAR-mediated EPSC amplitude at 1–30 min after addition of DHPG taken from **(C)** (*n* = 9 neurons from 7 *Cyld^+/+^* mice, *n* = 8 neurons from 4 *Cyld^−/−^* mice). **(F)** Absence of LTD induced by four trains of HFS (100 Hz, 1 s, with 10 s inter-train intervals) in field potential recordings in *Cyld^−/−^* mice. After HFS of the corticostriatal pathway, PS amplitude values in *Cyld^−/−^* mice were significantly higher than those in *Cyld^+/+^* mice between 1 and 60 min. **(G)** Representative traces showing the baseline (gray) and 1–60 min after LTD induction in *Cyld^+/+^* (black) and *Cyld^−/−^* (blue) mice. **(H)** Mean PS amplitude at 1–60 min after HFS taken from **(F)** (*n* = 16 slices from 10 *Cyld^+/+^* mice, *n* = 16 slices from 10 *Cyld^−/−^* mice). Data are presented as the mean ± SEM; *p < 0.05, **p < 0.01.

To elucidate whether CYLD is involved in the regulation of AMPAR removal, we examined the trafficking behavior of GluA1 and used immunohistochemical staining to analyze the amount of surface GluA1 on acute striatal slices with or without (RS)-3, 5-dihydroxyphenylglycine (DHPG) (100 μM, 10 min) treatment, which is known to activate mGluRs and trigger endocytosis of GluA1 (Zhang et al., 2008; Pick et al., 2017; Sanderson et al., 2018; van Gelder et al., 2020). First, we assessed the amount of surface protein using an antibody directed to the N-terminal domain (NTD) of GluA1, and found that GluA1 surface expression was significantly reduced in *Cyld^−/−^* mice under basal conditions, indicating that CYLD is involved in the regulation of GluA1 surface expression. Furthermore, we found that GluA1 surface expression in response to DHPG was significantly affected in *Cyld^−/−^* mice, further suggesting that CYLD contributes to DHPG-stimulated AMPAR trafficking (Figures 5A,B, interaction between genotype and treatment, *F*_(3, 57)_ = 13.390, *p* = 0.0006; main effect of genotype, *F*_(1, 57)_ = 3.135, *p* = 0.082; main effect of treatment, *F*_(1, 57)_ = 0.017, *p* = 0.898; *Cyld^+/+^*-Ctrl vs. *Cyld^−/−^*-Ctrl, *p* = 0.002; *Cyld^+/+^*-Ctrl vs. *Cyld^+/+^*-DHPG, *p* = 0.049; *Cyld^−/−^*-Ctrl vs. *Cyld^−/−^*-DHPG, *p* = 0.065; *Cyld^+/+^*-DHPG vs. *Cyld^−/−^*-DHPG, *p* = 0.541; two-way ANOVA with Tukey’s post-hoc test). This suggests that CYLD is essential for the removal of GluA1 by DHPG-induced mGluR activation.

### 3.6. CYLD deficiency impairs both DHPG-and HFS-induced LTD in the DLS

Synaptic plasticity is mainly a consequence of changes in synaptic transmission due to structural changes in the shape and number of spines. Glutamatergic synapses exhibit a long-term weakening of synaptic efficacy, known as LTD. The property and abundance of glutamate receptors play a crucial role in determining the impact of excitatory synaptic transmission and plasticity (Matsuzaki et al., 2004; Mabb and Ehlers, 2010; Maiti et al., 2015; Helm et al., 2021; Kasai et al., 2021). To further investigate whether impairment of DHPG-induced LTD in the DLS parallels the synaptic and molecular alterations noted above, LTD of excitatory corticostriatal transmission, induced by bath application of the mGluR agonist DHPG (100 μM, 10 min) (Zhu et al., 2018), was evaluated in whole-cell recordings. We found that DHPG-induced LTD deficits in *Cyld^−/−^* mice (Figures 5C,D, interaction between genotype and time, *F*_(39，580)_ = 1.881, *p* = 0.001; main effect of genotype, *F*_(1, 15)_ = 4.764, *p* = 0.045; main effect of time, *F*_(3.277, 48.74)_ = 4.403, *p* = 0.007, two-way repeated measures ANOVA; Figure 5E, *Cyld^+/+^*: 69.518 ± 4.457, *Cyld^−/−^*: 93.743 ± 10.779, t = 2.167, *p* = 0.047, unpaired Student’s *t*-test). These results are consistent with the hypothesis that defective GluA1 removal in *Cyld^−/−^* mice results in a DHPG-induced LTD deficit.

In the striatum, both DHPG-and HFS-induced LTD involve the activation of mGluRs and the removal of AMPARs (Sung et al., 2001; Skiteva et al., 2018). Therefore, we checked whether HFS-induced LTD is also impaired in DLS of *Cyld*^−/−^ mice. Using extracellular field potential recording of population spikes (PSs), we observed that CYLD deficiency impaired the induction and maintenance of HFS-induced field LTD in the corticostriatal pathway (Figures 5F,G, interaction between genotype and time, *F*_(73, 2,190)_ = 2.256, *p* < 0.0001; main effect of genotype, *F*_(1, 30)_ = 8.107, *p* = 0.008; main effect of time, *F*_(2.879, 86.38)_ = 14.680, *p* < 0.0001, two-way repeated measures ANOVA; Figure 5H, *Cyld^+/+^*: 80.320 ± 2.979, *Cyld^−/−^*: 90.860 ± 2.218, *t* = 2.837, p = 0.008, unpaired Student’s *t*-test).

## 4. Discussion

CYLD is associated with neuronal functions and circuits in the dorsal striatum (Zhang et al., 2016; Han et al., 2020). Here, we illustrate a new mechanism by which CYLD-mediated deubiquitination of GluA1 and GluA2 plays a constructive role in controlling excitatory synapse transmission and plasticity. CYLD specifically cleaves K63-linked polyUb chains, which has a linear topology of extended conformation and acts as a signal for sorting, trafficking and endocytosis (Mukhopadhyay and Riezman, 2007; Husnjak and Dikic, 2012; Foot et al., 2017). Our current study brings to light several striking aspects of CYLD function, expanding the understanding of how deubiquitination is involved in determining neuronal morphology and excitatory synaptic activity by regulating the stability and trafficking of AMPARs.

Dendritic spines, in which the PSD anchors glutamate receptors to the postsynaptic membrane, participate in excitatory neurotransmission and plasticity (Kasai et al., 2021). There is a tight correlation between spine shape and the functional expression of AMPARs (Matsuzaki et al., 2001), while levels of postsynaptic proteins involved in secretion and trafficking correlate poorly with synaptic strength in the immature (stubby) spine (Helm et al., 2021). Spine density and shape also depend on several factors, including age, neuronal cell type, and position along the dendrite (Herms and Dorostkar, 2016). CYLD is critical for promoting spine development and dendritic growth in cultured hippocampal neurons and CA1 hippocampal pyramidal neurons (Li et al., 2019; Colombo et al., 2021). Interestingly, CYLD deletion does not change MSN morphology in the striatum of 6-week-old mice (Colombo et al., 2021). In contrast, our results reveal that CYLD plays a critical role in regulating the morphological parameters of MSNs in the DLS of 12-to 20-week-old mice. CYLD deficiency results in a reduction in the complexity and extent of dendritic arborization accompanied by abnormal spine loss and dendritic beading, with attendant neuronal damage. Dendritic beading is an early hallmark of ongoing neuronal toxicity in a variety of pathological conditions, including traumatic brain injury, stroke-induced spreading depolarization, seizures, cytotoxic edema and mitochondrial dysfunction (Swann et al., 2000; Greenwood et al., 2007; Murphy et al., 2008; Sword et al., 2013). It appears that the transient or terminal nature of dendritic beading depends on the degree of calcium influx and mitochondrial depolarization inflicted by a neurotoxic challenge, which determines the ability of the neuron to recover its normal morphology (Greenwood et al., 2007). It is possible that CYLD deficiency may affect mitochondrial function and lead to neuronal toxicity, but this remains to be determined in future experiments. Moreover, stubby spines are viewed as an immature type based on their prevalence during early postnatal development and relative scarcity during brain maturation (Harris et al., 1992; Spacek and Harris, 1997; Berry and Nedivi, 2017; Helm et al., 2021), while mushroom spines are essential for adult brain function. The proportion of mushroom spines is significantly decreased, while the percentages of stubby spines and varicosity are increased in *Cyld^−/−^* MSNs (Figure 1). These data suggest that CYLD is not essential for the early stages of MSN spine development, i.e., spinogenesis, but plays a major role in spine refinement and maintenance in MSNs during postnatal development. In this sense, the mechanisms involved in CYLD-mediated spine development are different at the various neuronal developmental stages, and this should be investigated in future studies. In addition, CYLD deficiency leads to decreased MSN excitability as shown by increases in the rise time and half-width of APs, as well as a decrease in the AHP amplitude (Table 2).

Recent studies have shown a decrease in both the frequency of spontaneous excitatory postsynaptic currents and the amplitude of mEPSCs in principal neurons in the basolateral amygdala of *Cyld^−/−^* mice (Li et al., 2021). Similarly, we found that the amplitudes of both mEPSCs and AMPAR-mediated eEPSCs were reduced in *Cyld^−/−^* MSNs compared to *Cyld^+/+^* MSNs, suggesting a decrease in AMPAR levels at synapses. In addition, a lack of CYLD reduces the frequency of mEPSCs and increases the PPR of AMPAR-mediated eEPSCs, suggesting that CYLD regulates presynaptic glutamate release probability (Figure 2). In this study, sex as a biological variable did not affect the electrophysiological properties of MSNs. The above data indicate that CYLD is important for the modulation of AMPAR-mediated glutamatergic signaling in the DLS. The findings provide new knowledge regarding the neurobiological role of CYLD and further our understanding of AMPAR ubiquitination in neurophysiology.

Surface and synaptic expression of AMPARs in excitatory synapses is tightly regulated by ubiquitination, a posttranslational modification that regulates multiple aspects of AMPAR molecular biology including trafficking, localization and stability (Schwarz et al., 2010; Lussier et al., 2011, 2012; Widagdo et al., 2017). Ubiquitination of AMPARs by Nedd4-1 facilitates AMPAR endocytosis and trafficking to the lysosome, leading to a reduction in AMPAR surface localization and total receptor abundance, and consequently inhibition of synaptic transmission (Schwarz et al., 2010; Lin et al., 2011). The K63-ubiquitin chains are the primary posttranslational modification of GluA1 and GluA2 in various brain regions and the intracellular trafficking and degradation of GluA1 and GluA2 are ubiquitination-dependent (Huo et al., 2015; Widagdo et al., 2017); we uncovered in the current study that CYLD modulates the K63-linked polyUb chains of GluA1 and GluA2, which may subsequently affect AMPAR stability and trafficking in DLS. In the striatum, GluA1 endocytosis is thought to be important in the expression of LTD as triggered by DHPG-induced mGluR activation (Pick et al., 2017). Although GluA1 and GluA2 are also ubiquitination targets, the role of AMPAR ubiquitination in LTD remains poorly understood. In the current study, we found that CYLD interacts with GluA1 and GluA2 and CYLD deficiency is negatively correlated with the removal of GluA1 from the postsynaptic membrane. However, the detailed mechanisms underpinning how CYLD selects which modifier to target, how CYLD interacts with GluA1 and GluA2 and which subunit is more tightly associated with CYLD, remain to be uncovered.

It is worth noting that the effects observed in the conventional CYLD knockout mice used here could be due to compensatory effects resulting from long-term knockout. Alternatively, they could also come from global developmental defects in the brain resulting from the loss of CYLD. Thus, multiple mechanisms may exist that compensate for perturbations in certain cellular processes, and these mechanisms may only be recruited under special conditions (Zhu and Malinow, 2002). The chronic disabling of AMPAR function may trigger compensatory mechanisms for NMDAR-mediated synaptic transmission, which may be the reason why spine loss in *Cyld^−/−^* MSNs did not affect NMDAR-eEPSCs.

In summary, our results reveal the molecular basis that underlies CYLD regulation of striatal AMPAR function. CYLD deficiency causes an increase in K63-linked ubiquitination of GluA1 and GluA2, resulting in reduced GluA1 and GluA1 surface levels and therefore reduced AMPAR-dependent synaptic transmission in MSNs, which is associated with altered DHPG-and HFS-LTD. Thus, the present study identifies and characterizes CYLD substrates, which should provide insights into the molecular mechanisms of synapse organization, function and plasticity, as well as related neurodegenerative diseases.

## Data availability statement

The original contributions presented in the study are included in the article/Supplementary material, further inquiries can be directed to the corresponding authors.

## Ethics statement

This study was approved by the South China Normal University Institutional Review Boards. The use of animals in experiments was approved by the Institutional Animal Care and Use Committee (IACUC) and followed National Institutes of Health (NIH) guidelines.

## Author contributions

S-yT, LY, and CL designed the research. S-yT performed electrophysiological experiments, immunofluorescent staining, and data analysis. J-xJ contributed to biochemical experiments and statistical analysis. H-xH and J-rF performed part of data analysis of morphology. X-pM and YC performed cell culture and transfection. S-yT, LY, and CL drafted the manuscript, and all authors commented on it. All authors contributed to the article and approved the submitted version.

## Funding

This work was supported by the Natural Science Foundation of China (31871170, 32170950, and 31970915) and Natural Science Foundation of Guangdong Province (2021A1515010804).

## Conflict of interest

The authors declare that the research was conducted in the absence of any commercial or financial relationships that could be construed as a potential conflict of interest.

## Publisher’s note

All claims expressed in this article are solely those of the authors and do not necessarily represent those of their affiliated organizations, or those of the publisher, the editors and the reviewers. Any product that may be evaluated in this article, or claim that may be made by its manufacturer, is not guaranteed or endorsed by the publisher.

## References

[ref1] Bean B. P. (2007). The action potential in mammalian central neurons. Nat. Rev. Neurosci. 8, 451–465. doi: 10.1038/nrn2148 17514198

[ref2] Beattie E. C. Carroll R. C. Yu X. Morishita W. Yasuda H. Zastrow M. V.. (2000). Regulation of AMPAR receptor endocytosis by a signaling mechanism shared with LTD. Nat. Neurosci. 3, 12291–13000. 10.1038/81823 11100150

[ref3] Bello-Medina P. C. Flores G. Quirarte G. L. McGaugh J. L. Prado-Alcalá R. A. (2016). Mushroom spine dynamics in medium spiny neurons of dorsal striatum associated with memory of moderate and intense training. Proc. Natl. Acad. Sci. U. S. A. 113, E6516–E6525. doi: 10.1073/pnas.1613680113, PMID: 27698138 PMC5081589

[ref4] Berry K. P. Nedivi E. (2017). Spine dynamics: are they all the same? Neuron 96, 43–55. doi: 10.1016/j.neuron.2017.08.008, PMID: 28957675 PMC5661952

[ref5] Bignell G. Warren W. Seal S. Takahashi M. Rapley E. Barfoot R.. (2000). Identification of the familial cylindromatosis tumour-suppressor gene. Nat. Genet. 25, 160–165. doi: 10.1038/76006, PMID: 10835629

[ref6] Bredt D. S. Nicoll R. A. (2003). AMPA receptor trafficking at excitatory synapses. Neuron 40, 361–379. doi: 10.1016/S0896-6273(03)00640-8 14556714

[ref8] Chen M. Chen Y. Huo Q. Wang L. Tan S. Misrani A.. (2021). Enhancing GABAergic signaling ameliorates aberrant gamma oscillations of olfactory bulb in AD mouse models. Mol. Neurodegener. 16:14. doi: 10.1186/s13024-021-00434-7, PMID: 33663578 PMC7934466

[ref9] Chen M. Wang J. Z. Jiang J. X. Zheng X. Z. Justice N. J. Wang K.. (2017). APP modulates KCC2 expression and function in hippocampal GABAergic inhibition. elife 6:e20142. doi: 10.7554/eLife.20142, PMID: 28054918 PMC5224924

[ref10] Colombo E. Horta G. Roesler M. K. Ihbe N. Chhabra S. Radyushkin K.. (2021). The K63 deubiquitinase CYLD modulates autism-like behaviors and hippocampal plasticity by regulating autophagy and mTOR signaling. Proc. Natl. Acad. Sci. U. S. A. 118:e110755118. doi: 10.1073/pnas.2110755118 PMC8617491 34782467

[ref11] Crapser J. D. Ochaba J. Soni N. Reidling J. C. Thompson L. M. Green K. N. (2020). Microglial depletion prevents extracellular matrix changes and striatal volume reduction in a model of Huntington's disease. Brain 143, 266–288. doi: 10.1093/brain/awz363, PMID: 31848580 PMC6935750

[ref12] Diering G. H. Huganir R. L. (2018). The AMPA receptor code of synaptic plasticity. Neuron 100, 314–329. doi: 10.1016/j.neuron.2018.10.018, PMID: 30359599 PMC6214363

[ref13] Dobrunz L. E. Stevens C. F. (1997). Heterogeneity of release probability, facilitation, and depletion at central synapses. Neuron 18, 995–1008. doi: 10.1016/S0896-6273(00)80338-4, PMID: 9208866

[ref14] Dobson-Stone C. Hallupp M. Shahheydari H. Ragagnin A. M. G. Chatterton Z. Carew-Jones F.. (2020). CYLD is a causative gene for frontotemporal dementia - amyotrophic lateral sclerosis. Brain 143, 783–799. doi: 10.1093/brain/awaa039, PMID: 32185393 PMC7089666

[ref15] Dobson-Stone C. Luty A. A. Thompson E. M. Blumbergs P. Brooks W. S. Short C. L.. (2013). Frontotemporal dementia-amyotrophic lateral sclerosis syndrome locus on chromosome 16p12.1-q12.2: genetic, clinical and neuropathological analysis. Acta Neuropathol. 125, 523–533. doi: 10.1007/s00401-013-1078-9, PMID: 23338750 PMC3611035

[ref16] Dosemeci A. Thein S. Yang Y. Reese T. S. Tao-Cheng J. H. (2013). CYLD, a deubiquitinase specific for lysine 63-linked polyubiquitins, accumulates at the postsynaptic density in an activity-dependent manner. Biochem. Biophys. Res. Commun. 430, 245–249. doi: 10.1016/j.bbrc.2012.10.131, PMID: 23146630 PMC3545086

[ref17] Ferreira T. A. Blackman A. V. Oyrer J. Jayabal S. Chung A. J. Watt A. J.. (2014). Neuronal morphometry directly from bitmap images. Nat. Methods 11, 982–984. doi: 10.1038/nmeth.3125, PMID: 25264773 PMC5271921

[ref18] Foot N. Henshall T. Kumar S. (2017). Ubiquitination and the regulation of membrane protein. Physiol. Rev. 97, 23–281. doi: 10.1152/physrev.00012.2016 27932395

[ref19] Greenwood S. M. Mizielinska S. M. Frenguelli B. G. Harvey J. Connolly C. N. (2007). Mitochondrial dysfunction and dendritic beading during neuronal toxicity. J. Biol. Chem. 282, 26235–26244. doi: 10.1074/jbc.M704488200, PMID: 17616519

[ref20] Han Y. Y. Jin K. Pan Q. S. Li B. Wu Z. Q. Gan L.. (2020). Microglial activation in the dorsal striatum participates in anxiety-like behavior in Cyld knockout mice. Brain Behav. Immun. 89, 326–338. doi: 10.1016/j.bbi.2020.07.011, PMID: 32688031

[ref21] Harris K. M. Jensen F. E. Tsao B. (1992). Three-dimensional structure of dendritic spines and synapses in rat hippocampus (CA1) at postnatal day 15 and adult ages: implications for the maturation of synaptic physiology and long-term potentiation. J. Neurosci. 12, 2685–2705. doi: 10.1523/JNEUROSCI.12-07-02685.1992, PMID: 1613552 PMC6575840

[ref22] Helm M. S. Dankovich T. M. Mandad S. Rammner B. Jahne S. Salimi V.. (2021). A large-scale nanoscopy and biochemistry analysis of postsynaptic dendritic spines. Nat. Neurosci. 24, 1151–1162. doi: 10.1038/s41593-021-00874-w, PMID: 34168338

[ref23] Henley J. M. Wilkinson K. A. (2016). Synaptic AMPA receptor composition in development, plasticity and disease. Nat. Rev. Neurosci. 17, 337–350. doi: 10.1038/nrn.2016.37, PMID: 27080385

[ref24] Herms J. Dorostkar M. M. (2016). Dendritic spine pathology in neurodegenerative diseases. Annu. Rev. Pathol. 11, 221–250. doi: 10.1146/annurev-pathol-012615-044216 26907528

[ref25] Hoerndli F. J. Wang R. Mellem J. E. Kallarackal A. Brockie P. J. Thacker C.. (2015). Neuronal activity and CaMKII regulate kinesin-mediated transport of synaptic AMPARs. Neuron 86, 457–474. doi: 10.1016/j.neuron.2015.03.011, PMID: 25843407 PMC4409548

[ref26] Huganir R. L. Nicoll R. A. (2013). AMPARs and synaptic plasticity: the last 25 years. Neuron 80, 704–717. doi: 10.1016/j.neuron.2013.10.025, PMID: 24183021 PMC4195488

[ref27] Huo Y. Khatri N. Hou Q. Gilbert J. Wang G. Man H. Y. (2015). The deubiquitinating enzyme USP46 regulates AMPA receptor ubiquitination and trafficking. J. Neurochem. 134, 1067–1080. doi: 10.1111/jnc.13194, PMID: 26077708 PMC4668950

[ref28] Husnjak K. Dikic I. (2012). Ubiquitin-binding proteins: decoders of ubiquitin-mediated cellular functions. Annu. Rev. Biochem. 81, 291–322. doi: 10.1146/annurev-biochem-051810-094654, PMID: 22482907

[ref29] Isokawa M. (1997). Preservation of dendrites with the presence of reorganized mossy fiber collaterals in hippocampal dentate granule cells in patients with temporal lobe epilepsy. Brain Res. 744, 339–343. doi: 10.1016/S0006-8993(96)01067-0, PMID: 9027394

[ref30] Jiang J. Tang B. Wang L. Huo Q. Tan S. Misrani A.. (2022). Systemic LPS-induced microglial activation results in increased GABAergic tone: a mechanism of protection against neuroinflammation in the medial prefrontal cortex in mice. Brain Behav. Immun. 99, 53–69. doi: 10.1016/j.bbi.2021.09.017, PMID: 34582995

[ref31] Kanner S. A. Shuja Z. Choudhury P. Jain A. Colecraft H. M. (2020). Targeted deubiquitination rescues distinct trafficking-deficient ion channelopathies. Nat. Methods 17, 1245–1253. doi: 10.1038/s41592-020-00992-6, PMID: 33169015 PMC9335257

[ref32] Kantamneni S. Wilkinson K. A. Henley J. M. (2011). Ubiquitin regulation of neuronal excitability. Nat. Neurosci. 14, 126–128. doi: 10.1038/nn0211-126, PMID: 21270777 PMC3308140

[ref33] Kasai H. Ziv N. E. Okazaki H. Yagishita S. Toyoizumi T. (2021). Spine dynamics in the brain, mental disorders and artificial neural networks. Nat. Rev. Neurosci. 22, 407–422. doi: 10.1038/s41583-021-00467-3, PMID: 34050339

[ref34] Knackstedt L. A. Trantham-Davidson H. L. Schwendt M. (2014). The role of ventral and dorsal striatum mGluR5 in relapse to cocaine-seeking and extinction learning. Addict. Biol. 19, 87–101. doi: 10.1111/adb.12061, PMID: 23710649 PMC3762937

[ref35] Li H. D. Li D. N. Yang L. Long C. (2021). Deficiency of the CYLD impairs fear memory of mice and disrupts neuronal activity and synaptic transmission in the basolateral amygdala. Front. Cell. Neurosci. 15:740165. doi: 10.3389/fncel.2021.740165, PMID: 34602983 PMC8485066

[ref36] Li J. Sekine-Aizawa Y. Ebrahimi S. Tanaka S. Okabe S. (2019). Tumor suppressor protein CYLD regulates morphogenesis of dendrites and spines. Eur. J. Neurosci. 50, 2722–2739. doi: 10.1111/ejn.14421, PMID: 31001844

[ref37] Li X. Zhang Z. Zhang Y. Cao Y. Wei H. Wu Z. (2018). Upregulation of lactate-inducible snail protein suppresses oncogene-mediated senescence through p16 (INK4a) inactivation. J. Exp. Clin. Cancer Res. 37:39. doi: 10.1186/s13046-018-0701-y, PMID: 29482580 PMC5828408

[ref38] Lin A. Hou Q. Jarzylo L. Amato S. Gilbert J. Shang F.. (2011). Nedd4-mediated AMPA receptor ubiquitination regulates receptor turnover and trafficking. J. Neurochem. 119, 27–39. doi: 10.1111/j.1471-4159.2011.07221.x, PMID: 21338354 PMC3110981

[ref39] Llano O. Smirnov S. Soni S. Golubtsov A. Guillemin I. Hotulainen P.. (2015). KCC2 regulates actin dynamics in dendritic spines via interaction with beta-PIX. J. Cell Biol. 209, 671–686. doi: 10.1083/jcb.201411008, PMID: 26056138 PMC4460141

[ref40] Luscher C. Huber K. M. (2010). Group 1 mGluR-dependent synaptic long-term depression: mechanisms and implications for circuitry and disease. Neuron 65, 445–459. doi: 10.1016/j.neuron.2010.01.016, PMID: 20188650 PMC2841961

[ref41] Lussier M. P. Herring B. E. Nasu-Nishimura Y. Neutzner A. Karbowski M. Youle R. J.. (2012). Ubiquitin ligase RNF167 regulates AMPA receptor-mediated synaptic transmission. Proc. Natl. Acad. Sci. U. S. A. 109, 19426–19431. doi: 10.1073/pnas.1217477109, PMID: 23129617 PMC3511152

[ref42] Lussier M. P. Nasu-Nishimura Y. Roche K. W. (2011). Activity-dependent ubiquitination of the AMPA receptor subunit Glu A2. J. Neurosci. 31, 3077–3081. doi: 10.1523/JNEUROSCI.5944-10.2011, PMID: 21414928 PMC3081723

[ref43] Ma Q. Ruan H. Peng L. Zhang M. Gack M. U. Yao W. D. (2017). Proteasome-independent polyubiquitin linkage regulates synapse scaffolding, efficacy, and plasticity. Proc. Natl. Acad. Sci. U. S. A. 114, E8760–E8769. doi: 10.1073/pnas.1620153114, PMID: 28973854 PMC5642675

[ref44] Mabb A. M. Ehlers M. D. (2010). Ubiquitination in postsynaptic function and plasticity. Annu. Rev. Cell Dev. Biol. 26, 179–210. doi: 10.1146/annurev-cellbio-100109-104129, PMID: 20604708 PMC3163670

[ref45] Maiti P. Manna J. Ilavazhagan G. Rossignol J. Dunbar G. L. (2015). Molecular regulation of dendritic spine dynamics and their potential impact on synaptic plasticity and neurological diseases. Neurosci. Biobehav. Rev. 59, 208–237. doi: 10.1016/j.neubiorev.2015.09.020, PMID: 26562682

[ref46] Mao M. Nair A. Augustine G. J. (2019). A novel type of neuron within the dorsal striatum. Front. Neural Circuits 13:32. doi: 10.3389/fncir.2019.00032, PMID: 31164808 PMC6536632

[ref47] Matsuzaki M. Ellis-Davies G. C. Nemoto T. Miyashita Y. Iino M. Kasai H. (2001). Dendritic spine geometry is critical for AMPA receptor expression in hippocampal CA1 pyramidal neurons. Nat. Neurosci. 4, 1086–1092. doi: 10.1038/nn736, PMID: 11687814 PMC4229049

[ref48] Matsuzaki M. Honkura N. Ellis-Davies G. C. Kasai H. (2004). Structural basis of long-term potentiation in single dendritic spines. Nature 429, 761–766. doi: 10.1038/nature02617, PMID: 15190253 PMC4158816

[ref49] Mazarei G. Neal S. J. Becanovic K. Luthi-Carter R. Simpson E. M. Leavitt B. R. (2010). Expression analysis of novel striatal-enriched genes in Huntington disease. Hum. Mol. Genet. 19, 609–622. doi: 10.1093/hmg/ddp527, PMID: 19934114 PMC2807369

[ref50] Mei Y. Monteiro P. Zhou Y. Kim J. A. Gao X. Fu Z.. (2016). Adult restoration of Shank 3 expression rescues selective autistic-like phenotypes. Nature 530, 481–484. doi: 10.1038/nature16971, PMID: 26886798 PMC4898763

[ref51] Misrani A. Tabassum S. Chen X. Tan S. Y. Wang J. C. Yang L.. (2019). Differential effects of citalopram on sleep-deprivation-induced depressive-like behavior and memory impairments in mice. Prog. Neuro Psychopharmacol. Biol. Psychiatry 88, 102–111. doi: 10.1016/j.pnpbp.2018.07.013, PMID: 30017777

[ref52] Moussawi K. Pacchioni A. Moran M. Olive M. F. Gass J. T. Lavin A.. (2009). N-acetylcysteine reverses cocaine-induced metaplasticity. Nat. Neurosci. 12, 182–189. doi: 10.1038/nn.2250, PMID: 19136971 PMC2661026

[ref53] Mukhopadhyay D. Riezman H. (2007). Proteasome-independent functions of ubiquitin in endocytiosis and signaling. Science 315, 201–205. doi: 10.1126/science.1127085, PMID: 17218518

[ref54] Murphy T. H. Li P. Betts K. Liu R. (2008). Two-photon imaging of stroke onset in vivo reveals that NMDA-receptor independent ischemic depolarization is the major cause of rapid reversible damage to dendrites and spines. J. Neurosci. 28, 1756–1772. doi: 10.1523/JNEUROSCI.5128-07.2008, PMID: 18272696 PMC6671530

[ref55] Nazzaro C. Greco B. Cerovic M. Baxter P. Rubino T. Trusel M.. (2012). SK channel modulation rescues striatal plasticity and control over habit in cannabinoid tolerance. Nat. Neurosci. 15, 284–293. doi: 10.1038/nn.3022, PMID: 22231426

[ref56] Opazo P. Labrecque S. Tigaret C. M. Frouin A. Wiseman P. W. De Koninck P.. (2010). CaMKII triggers the diffusional trapping of surface AMPARs through phosphorylation of stargazin. Neuron 67, 239–252. doi: 10.1016/j.neuron.2010.06.007, PMID: 20670832

[ref57] Palazon-Riquelme P. Worboys J. D. Green J. Valera A. Martin-Sanchez F. Pellegrini C.. (2018). USP7 and USP47 deubiquitinases regulate NLRP3 inflammasome activation. EMBO Rep. 19, 253–281. doi: 10.15252/embr.201744766, PMID: 30206189 PMC6172458

[ref58] Peng Y. G. Cai P. J. Hu J. H. Jiang J. X. Zhang J. J. Liu K. F.. (2021). Altered corticostriatal synchronization associated with compulsive-like behavior in APP/PS1 mice. Exp. Neurol. 344:113805. doi: 10.1016/j.expneurol.2021.113805, PMID: 34242631

[ref59] Pick J. E. Khatri L. Sathler M. F. Ziff E. B. (2017). mGluR long-term depression regulates GluA2 association with COPII vesicles and exit from the endoplasmic reticulum. EMBO J. 36, 232–244. doi: 10.15252/embj.201694526, PMID: 27856517 PMC5239995

[ref60] Popescu G. Auerbach A. (2003). Modal gating of NMDA receptors and the shape of their synaptic response. Nat. Neurosci. 6, 476–483. doi: 10.1038/nn1044, PMID: 12679783

[ref61] Reiley W. W. Jin W. Lee A. J. Wright A. Wu X. Tewalt E. F.. (2007). Deubiquitinating enzyme CYLD negatively regulates the ubiquitin-dependent kinase Tak1 and prevents abnormal T cell responses. J. Exp. Med. 204, 1475–1485. doi: 10.1084/jem.20062694, PMID: 17548520 PMC2118606

[ref63] Sanderson T. M. Bradley C. A. Georgiou J. Hong Y. H. Ng A. N. Lee Y.. (2018). The probability of neurotransmitter release governs AMPA receptor trafficking via activity-dependent regulation of mGluR1 surface expression. Cell Rep. 25, 3631–3646.e3. doi: 10.1016/j.celrep.2018.12.010, PMID: 30590038 PMC6315206

[ref64] Santos S. D. Carvalho A. L. Caldeira M. V. Duarte C. B. (2009). Regulation of AMPA receptors and synaptic plasticity. Neuroscience 158, 105–125. doi: 10.1016/j.neuroscience.2008.02.037 18424006

[ref65] Sato Y. Goto E. Shibata Y. Kubota Y. Yamagata A. Goto-Ito S.. (2015). Structures of CYLD USP with Met 1-or Lys63-linked diubiquitin reveal mechanisms for dual specificity. Nat. Struct. Mol. Biol. 22, 222–229. doi: 10.1038/nsmb.2970, PMID: 25686088

[ref66] Schwarz L. A. Hall B. J. Patrick G. N. (2010). Activity-dependent ubiquitination of GluA1 mediates a distinct AMPA receptor endocytosis and sorting pathway. J. Neurosci. 30, 16718–16729. doi: 10.1523/JNEUROSCI.3686-10.2010, PMID: 21148011 PMC3079366

[ref67] Skiteva O. Yao N. Nouhi M. Chergui K. (2018). High frequency stimulation induces LTD of AMPA receptor-mediated postsynaptic responses and LTP of synaptically-evoked firing in the dorsolateral striatum. Neurosci. Lett. 666, 11–16. doi: 10.1016/j.neulet.2017.12.028, PMID: 29248613

[ref68] Spacek J. Harris K. M. (1997). Three-dimensional organization of smooth endoplasmic reticulum in hippocampal CA1 dendrites and dendritic spines of the immature and mature rat. J. Neurosci. 17, 190–203. doi: 10.1523/JNEUROSCI.17-01-00190.1997, PMID: 8987748 PMC6793680

[ref69] Sung K. W. Choi S. Lovinger D. M. (2001). Activation of group I mGluRs is necessary for induction of long-term depression at striatal synapses. J. Neurophysiol. 86, 2405–2412. doi: 10.1152/jn.2001.86.5.2405, PMID: 11698530

[ref70] Swann J. W. Al-Noori S. Jiang M. Lee C. L. (2000). Spine loss and other dendritic abnormalities in epilepsy. Hippocampus 10, 617–625. doi: 10.1002/1098-1063(2000)10:5<617::AID-HIPO13>3.0.CO;2-R, PMID: 11075833

[ref71] Sword J. Masuda T. Croom D. Kirov S. A. (2013). Evolution of neuronal and astroglial disruption in the peri-contusional cortex of mice revealed by in vivo two-photon imaging. Brain 136, 1446–1461. doi: 10.1093/brain/awt026, PMID: 23466395 PMC3634194

[ref72] Tabuas-Pereira M. Santana I. Kun-Rodrigues C. Bras J. Guerreiro R. (2020). CYLD variants in frontotemporal dementia associated with severe memory impairment in a Portuguese cohort. Brain 143:e67. doi: 10.1093/brain/awaa183, PMID: 32666117

[ref73] van Gelder C. Penning R. Veth T. S. Catsburg L. A. E. Hoogenraad C. C. MacGillavry H. D.. (2020). Temporal quantitative proteomics of mGluR-induced protein translation and phosphorylation in neurons. Mol. Cell. Proteomics 19, 1952–1968. doi: 10.1074/mcp.RA120.002199, PMID: 32912969 PMC7710149

[ref74] Wang S. Tanzi R. E. Li A. (2019). Quantitative analysis of neuronal dendritic arborization complexity in drosophila. J. Vis. Exp. 143:e57139. doi: 10.3791/57139 30663659

[ref75] Westrum L. E. White L. E. Jr. Ward A. A. Jr. (1964). Morphology of the experimental epileptic focus. J. Neurosurg. 21, 1033–1046. doi: 10.3171/jns.1964.21.12.1033 14279823

[ref76] Widagdo J. Guntupalli S. Jang S. E. Anggono V. (2017). Regulation of AMPA receptor trafficking by protein ubiquitination. Front. Mol. Neurosci. 10:347. doi: 10.3389/fnmol.2017.00347, PMID: 29123470 PMC5662755

[ref77] Yang S. Ma N. Wu X. Ni H. Gao S. Sun L.. (2021). CYLD deficiency causes auditory neuropathy due to reduced neurite outgrowth. J. Clin. Lab. Anal. 35:e23783. doi: 10.1002/jcla.23783, PMID: 33934395 PMC8183908

[ref78] Yasuda H. Yamamoto H. Hanamura K. Mehruba M. Kawamata T. Morisaki H.. (2020). PKN1 promotes synapse maturation by inhibiting mGluR-dependent silencing through neuronal glutamate transporter activation. Commun. Biol. 3:710. doi: 10.1038/s42003-020-01435-w, PMID: 33244074 PMC7691520

[ref79] Yu J. Rao P. Clark S. Mitra J. Ha T. Gouaux E. (2021). Hippocampal AMPA receptor assemblies and mechanism of allosteric inhibition. Nature 594, 448–453. doi: 10.1038/s41586-021-03540-0, PMID: 33981040 PMC8270219

[ref80] Zajicek A. S. Ruan H. Dai H. Skolfield M. C. Phillips H. L. Burnette W. J.. (2022). Cylindromatosis drives synapse pruning and weakening by promoting macroautophagy through Akt-mTOR signaling. Mol. Psychiatry 27, 2414–2424. doi: 10.1038/s41380-022-01571-1, PMID: 35449295 PMC9278694

[ref81] Zhang J. Chen M. Li B. Lv B. Jin K. Zheng S.. (2016). Altered striatal rhythmic activity in cylindromatosis knock-out mice due to enhanced GABAergic inhibition. Neuropharmacology 110, 260–267. doi: 10.1016/j.neuropharm.2016.06.021, PMID: 27342122

[ref82] Zhang Y. Venkitaramani D. V. Gladding C. M. Zhang Y. Kurup P. Molnar E.. (2008). The tyrosine phosphatase STEP mediates AMPA receptor endocytosis after metabotropic glutamate receptor stimulation. J. Neurosci. 28, 10561–10566. doi: 10.1523/JNEUROSCI.2666-08.2008, PMID: 18923032 PMC2586105

[ref83] Zhang D. Y. Watson J. F. Matthews P. M. Cais O. Greger I. H. (2021). Gating and modulation of a hetero-octameric AMPA glutamate receptor. Nature 594, 454–458. doi: 10.1038/s41586-021-03613-0, PMID: 34079129 PMC7611729

[ref84] Zhou Z. Liu A. Xia S. Leung C. Qi J. Meng Y.. (2018). The C-terminal tails of endogenous GluA1 and GluA2 differentially contribute to hippocampal synaptic plasticity and learning. Nat. Neurosci. 21, 50–62. doi: 10.1038/s41593-017-0030-z, PMID: 29230056

[ref85] Zhu P. J. Chen C. J. Mays J. Stoica L. Costa-Mattioli M. (2018). mTORC2, but not mTORC1, is required for hippocampal mGluR-LTD and associated behaviors. Nat. Neurosci. 21, 799–802. doi: 10.1038/s41593-018-0156-7, PMID: 29786082 PMC6467217

[ref86] Zhu J. J. Malinow R. (2002). Acute versus chronic NMDA receptor blockade and synaptic AMPA receptor delivery. Nat. Neurosci. 5, 513–514. doi: 10.1038/nn0602-850, PMID: 11967548

[ref87] Zinebi F. Russell R. T. McKernan M. Shinnick-Gallagher P. (2001). Comparison of paired-pulse facilitation of AMPA and NMDA synaptic currents in the lateral amygdala. Synapse 42, 115–127. doi: 10.1002/syn.1107, PMID: 11574948

