# Corrigendum: Neural mechanism underlies CYLD modulation of morphology and synaptic function of medium spiny neurons in dorsolateral striatum

**DOI:** 10.3389/fnmol.2023.1162275

**Published:** 2023-02-23

**Authors:** Shu-yi Tan, Jin-xiang Jiang, Hui-xian Huang, Xiu-ping Mo, Jing-ru Feng, Yu Chen, Li Yang, Cheng Long

**Affiliations:** ^1^School of Life Sciences, South China Normal University, Guangzhou, China; ^2^School of Life Sciences, Guangzhou University, Guangzhou, China; ^3^South China Normal University-Panyu Central Hospital Joint Laboratory of Translational Medical Research, Panyu Central Hospital, Guangzhou, China

**Keywords:** CYLD, AMPAR, GluA1, GluA2, K63-linked ubiquitination, synaptic transmission, long-term depression, dorsolateral striatum

In the published article, there was an error in the legend for “Figure 1” as published. “**(H)** Bar graphs showing more stubby spines and varicosity, but fewer mushroom spines and filopodia, in *Cyld*^−/−^ MSNs than in *Cyld*^+/+^ MSNs.” The corrected legend appears below.

“**(H)** Bar graphs showing more stubby spines and varicosity, but fewer mushroom spines, in *Cyld*^−/−^ MSNs than in *Cyld*^+/+^ MSNs.”

In the published article, there was an error. “*P*-value error for the main effect of distance in Figure 1E.”

A correction has been made to section **3. Results**, “*3.1. CYLD affects the morphology and physiological features of MSNs*,” [Paragraph 1]. This sentence previously stated:

“main effect of distance, *F*_(3.202,153.3)_ = 202.200, *p* = 0.188”

The corrected sentence appears below:

“main effect of distance, *F*_(3.202,153.3)_ = 202.200, *p* < 0.0001”

In the published article, there was an error. “Misspelling on a word “GluA2”.”

A correction has been made to section **4. Discussion**, “The last paragraph.” This sentence previously stated:

“CYLD deficiency causes an increase in K63-linked ubiquitination of GluA1 and GluA2, resulting in reduced GluA1 and GluA1 surface levels and therefore reduced AMPAR-dependent synaptic transmission in MSNs, which is associated with altered DHPG-and HFS-LTD.”

The corrected sentence appears below:

“CYLD deficiency causes an increase in K63-linked ubiquitination of GluA1 and GluA2, resulting in reduced GluA1 and GluA2 surface levels and therefore reduced AMPAR-dependent synaptic transmission in MSNs, which is associated with altered DHPG-and HFS-LTD.”

The authors apologize for this error and state that this does not change the scientific conclusions of the article in any way. The original article has been updated.

