# Supplementary figures and images for: Neural mechanism underlies CYLD modulation of morphology and synaptic function of medium spiny neurons in dorsolateral striatum
Source: Front Mol Neurosci. 2023 Feb 8;16:1107355. doi: 10.3389/fnmol.2023.1107355 (PMC9945542; doi:10.3389/fnmol.2023.1107355)

Figure 1B

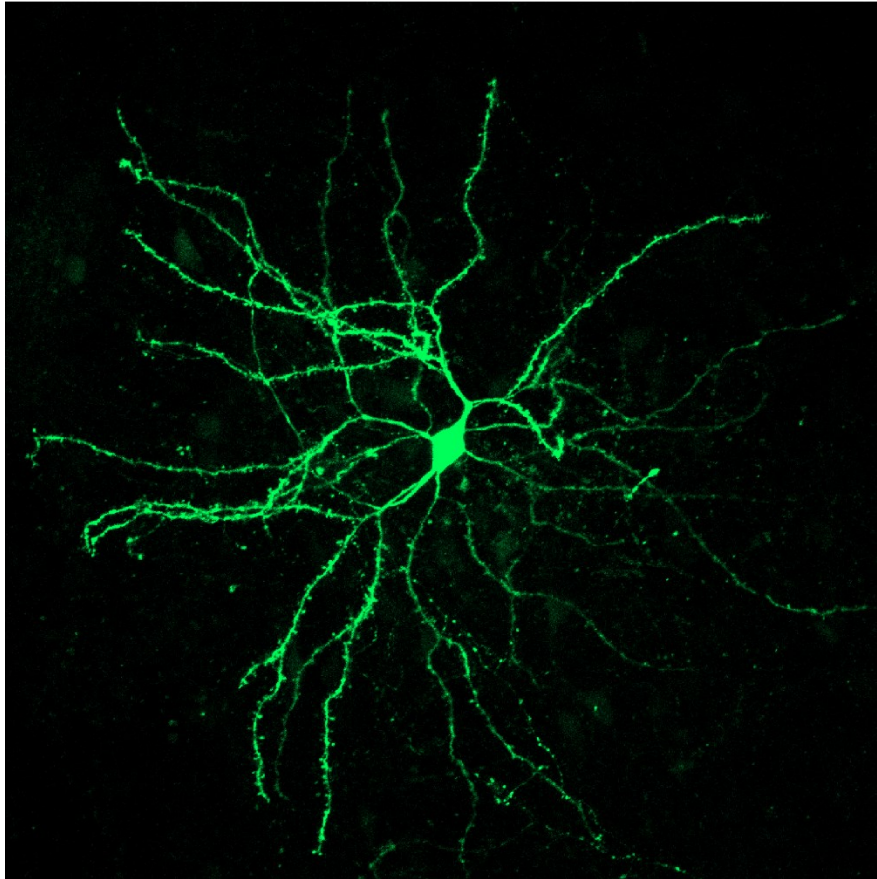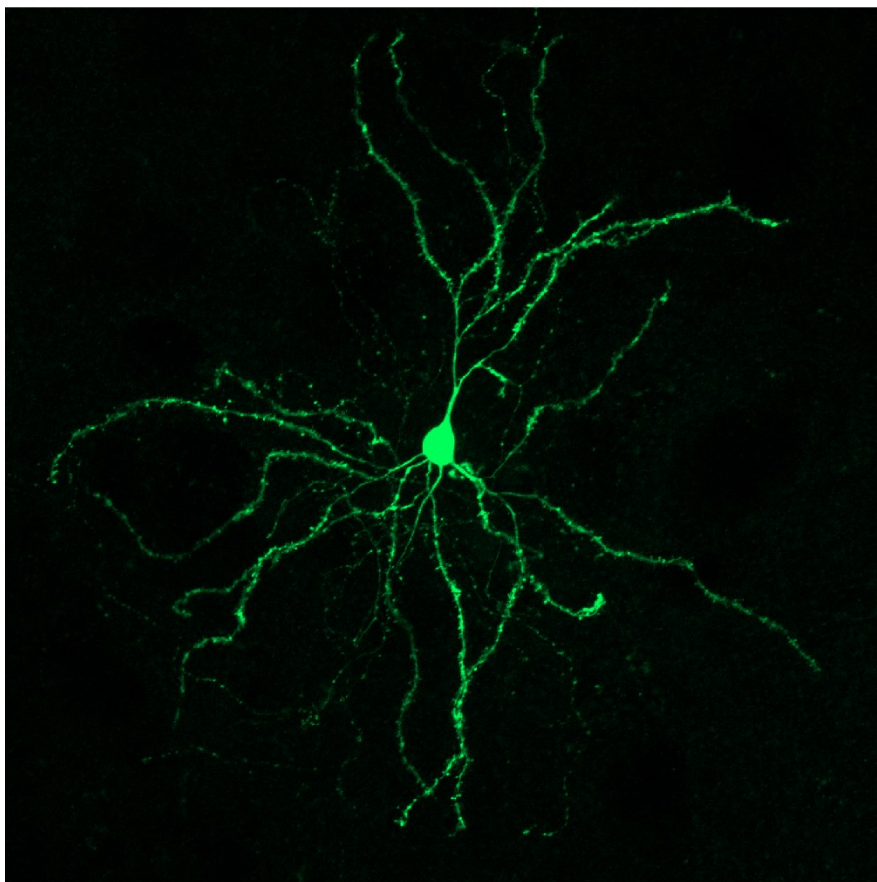

Figure 1F

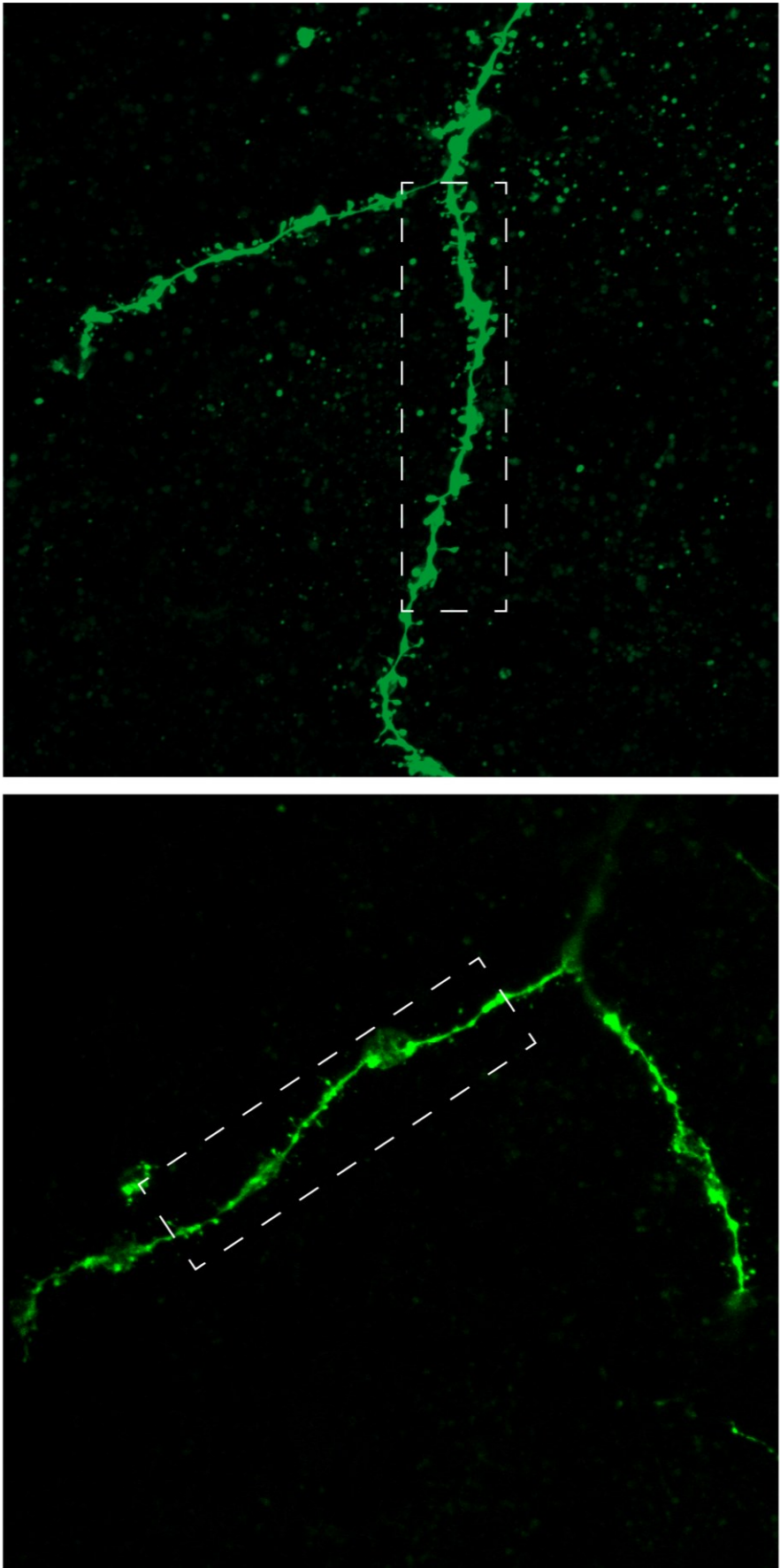

Supplement: Supplementary file 1 [file Data_Sheet_1.pdf]
