# Supplementary material for: Neural mechanism underlies CYLD modulation of morphology and synaptic function of medium spiny neurons in dorsolateral striatum
Source: Front Mol Neurosci. 2023 Feb 8;16:1107355. doi: 10.3389/fnmol.2023.1107355 (PMC9945542; doi:10.3389/fnmol.2023.1107355)
Supplement: Supplementary file 2 [file Data_Sheet_2.pdf]

Figure 3A

1.CYLD

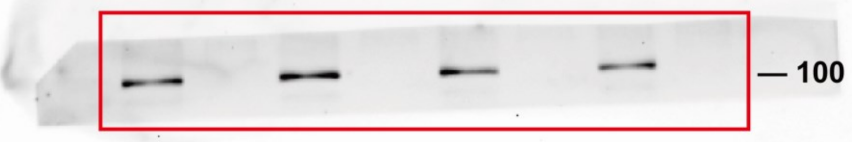

2.GluA1

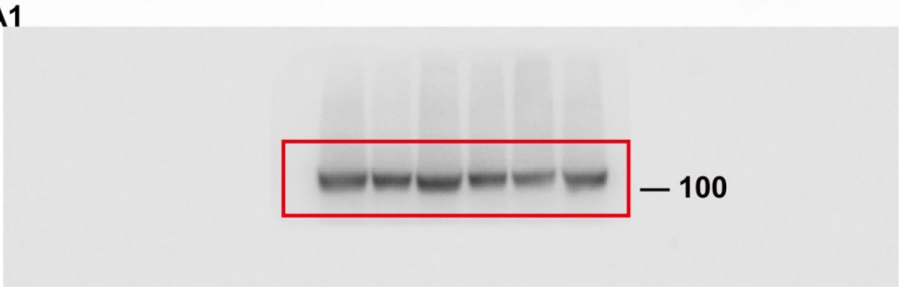

3.GluA2

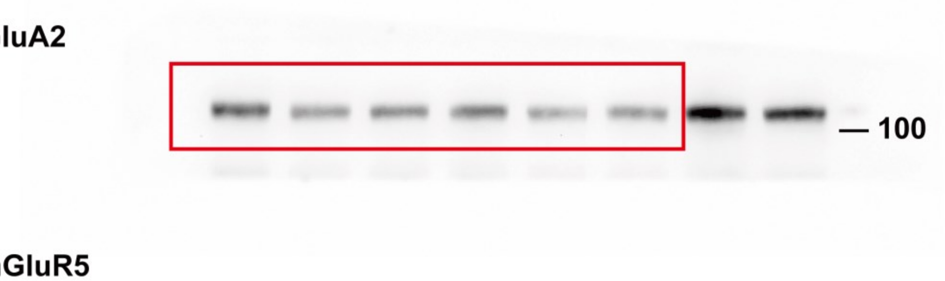

4.mGluR5

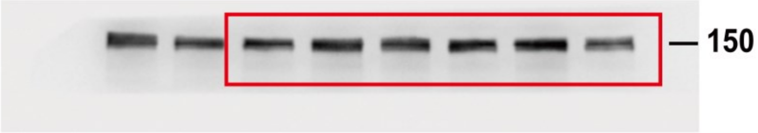

5.CaMKII

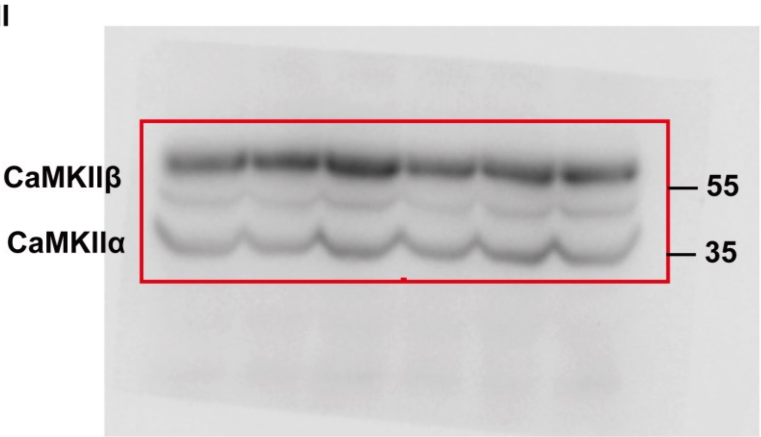

6.pCaMKII

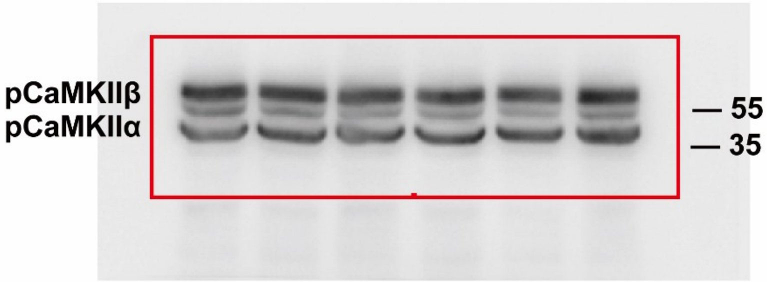

7.β-actin

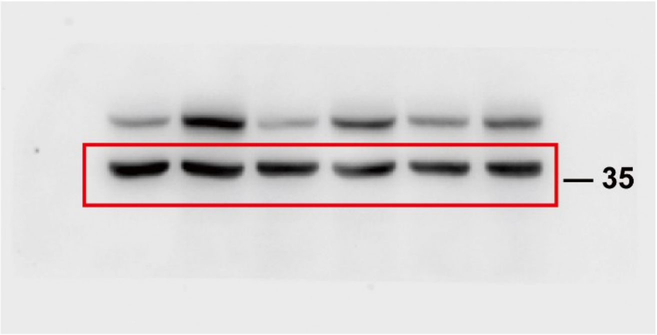

Figure 3C

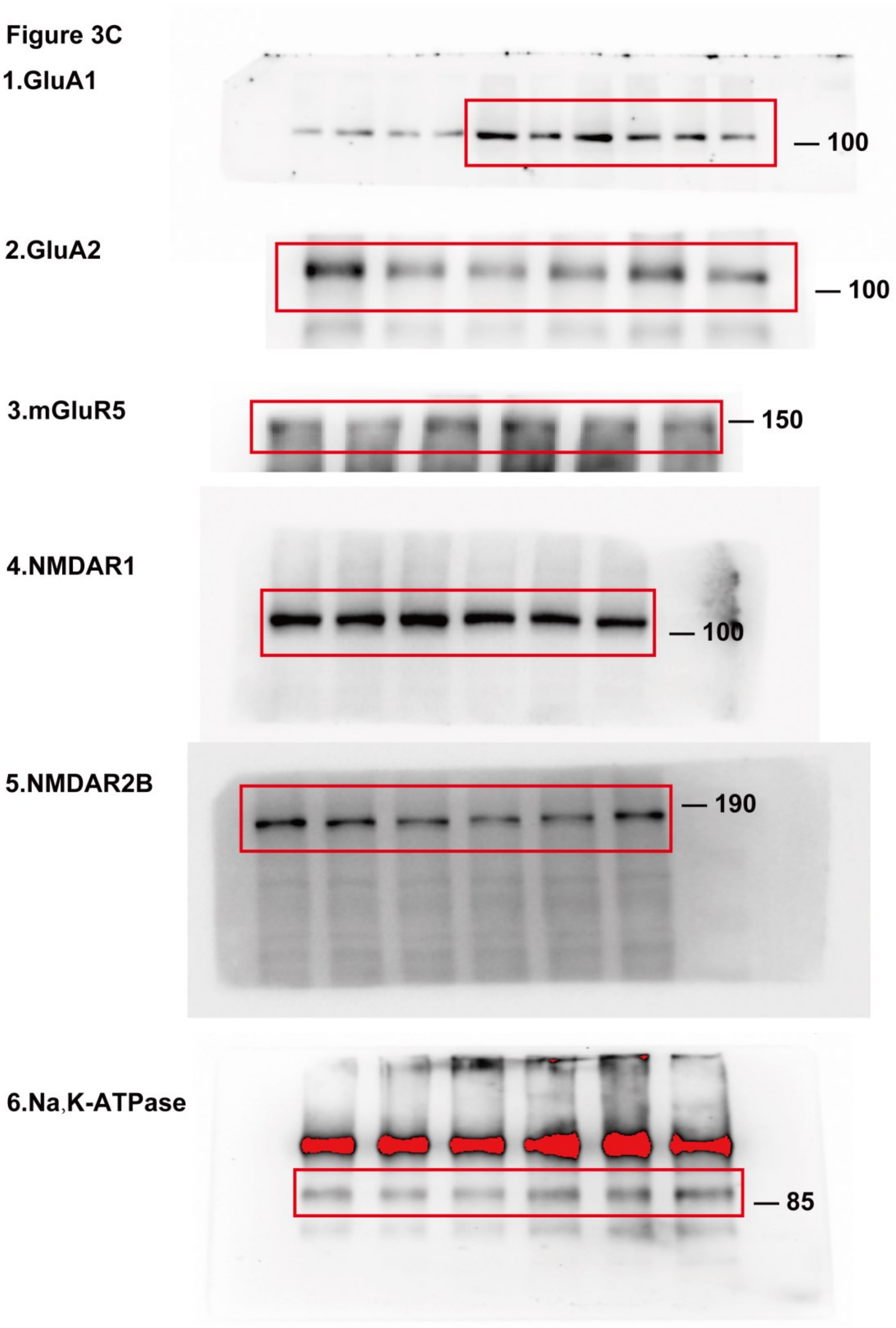
1.GluA1
100
2.GluA2
100
3.mGluR5
150
4.NMDAR1
100
5.NMDAR2B
190
6.Na,K-ATPase
85
