# Supplementary material for: Neural mechanism underlies CYLD modulation of morphology and synaptic function of medium spiny neurons in dorsolateral striatum
Source: Front Mol Neurosci. 2023 Feb 8;16:1107355. doi: 10.3389/fnmol.2023.1107355 (PMC9945542; doi:10.3389/fnmol.2023.1107355)

Figure 4A

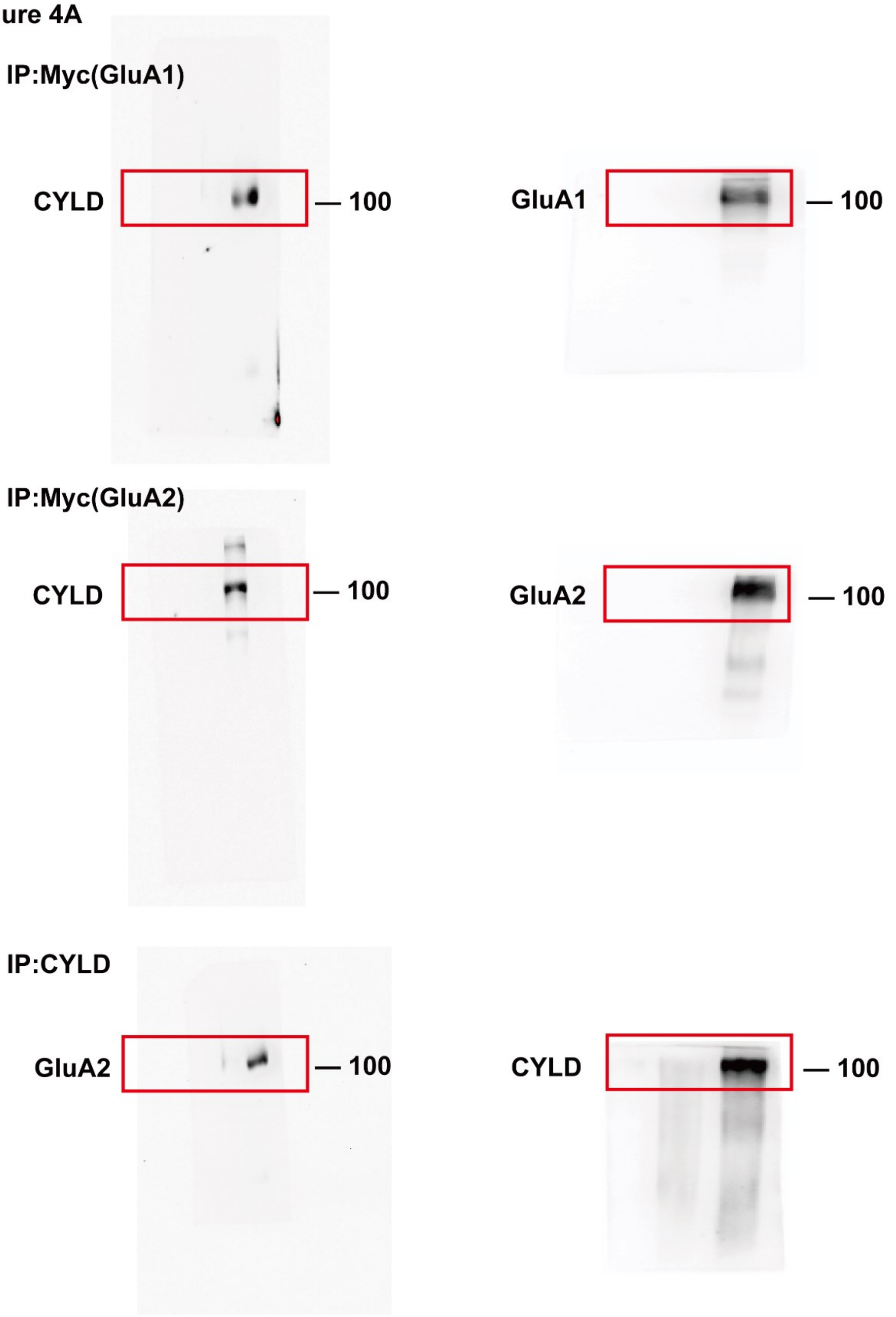
IP:Myc(GluA1)
CYLD
— 100
GluA1
— 100
IP:Myc(GluA2)
CYLD
— 100
GluA2
— 100
IP:CYLD
GluA2
— 100
CYLD
— 100

Figure 4B

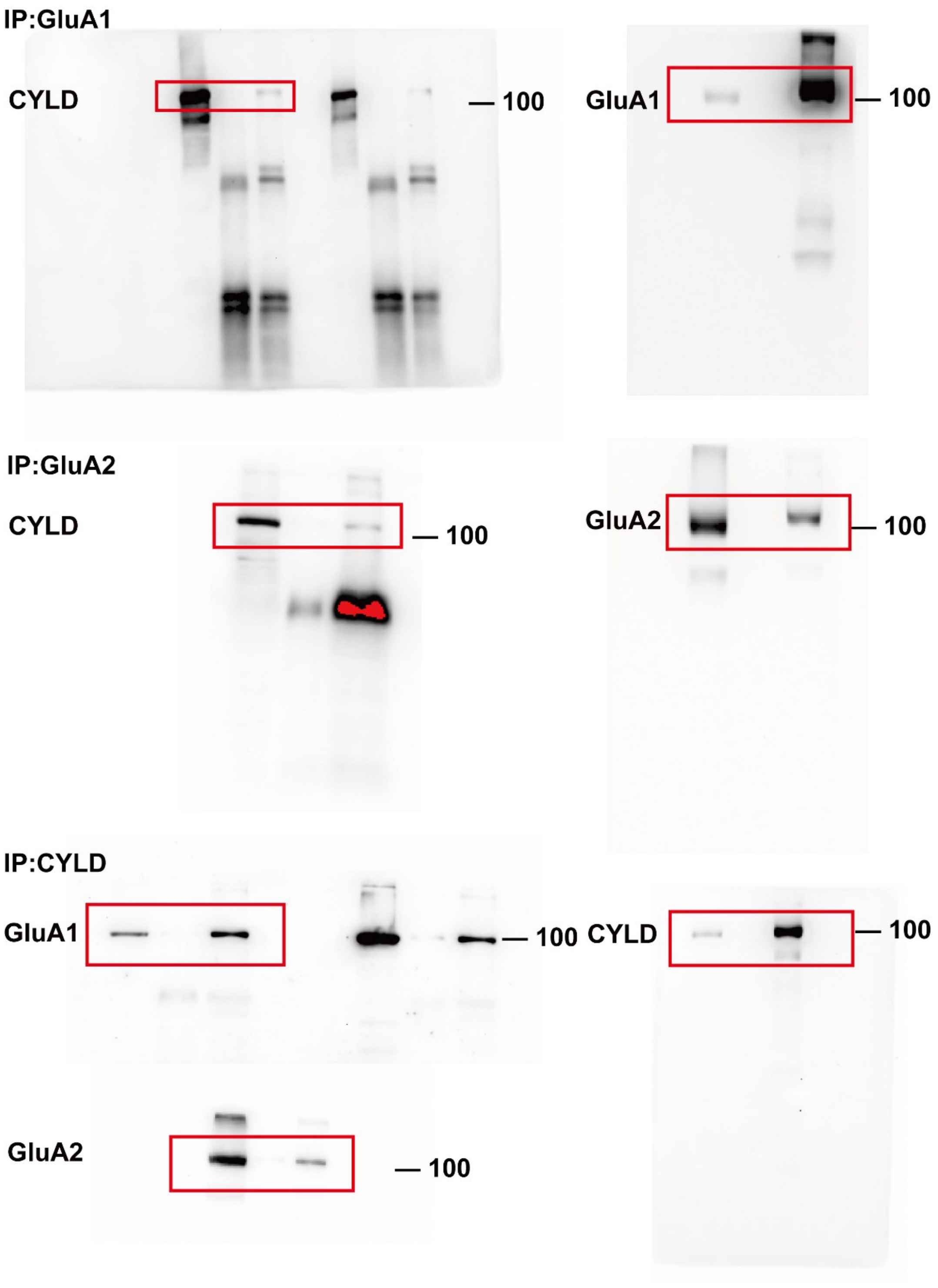
IP:GluA1
CYLD
— 100
GluA1
— 100
IP:GluA2
CYLD
— 100
GluA2
— 100
IP:CYLD
GluA1
— 100
CYLD
— 100
GluA2
— 100

Figure 4C

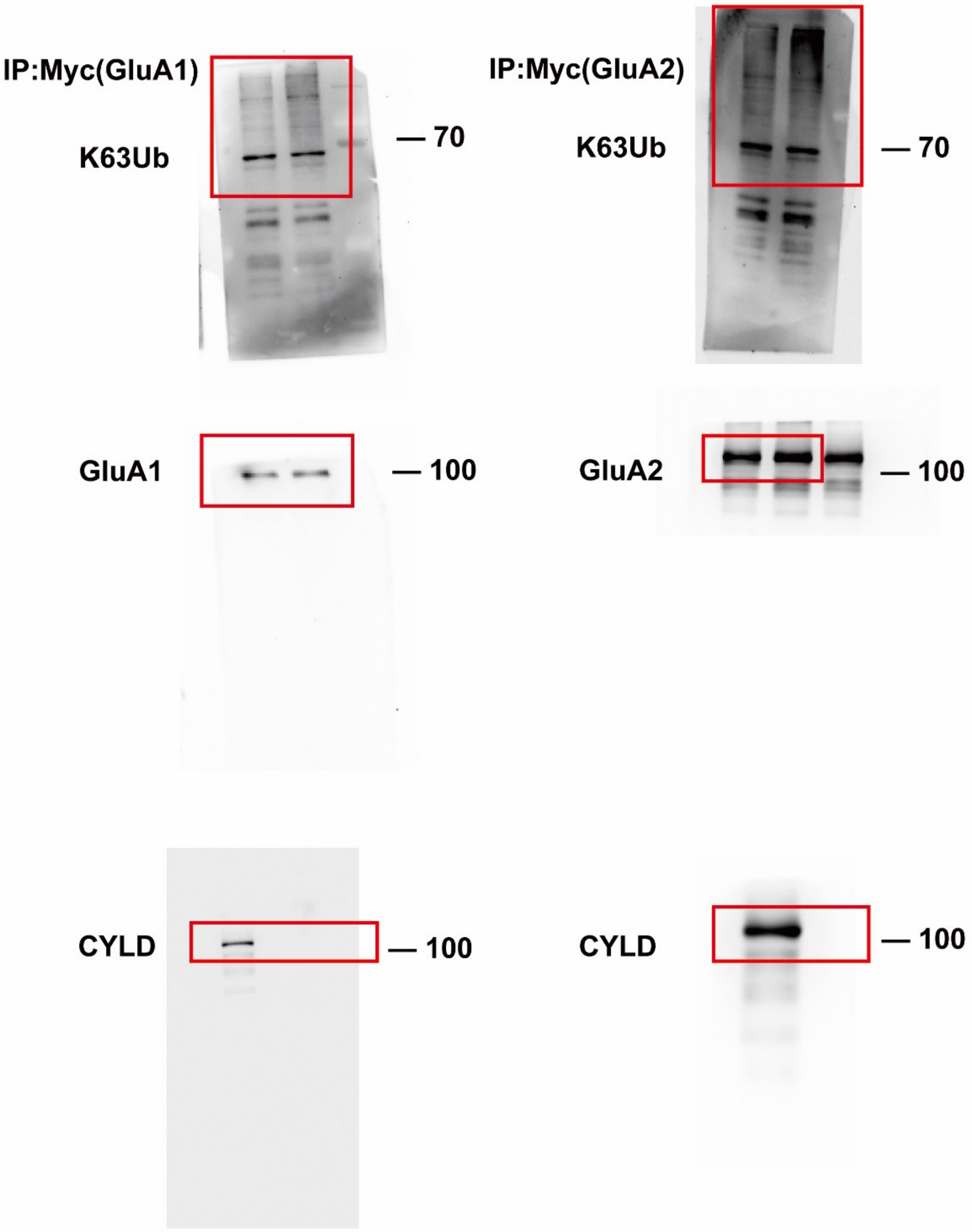
IP:Myc(GluA1)
K63Ub
70
GluA1
100
CYLD
100
IP:Myc(GluA2)
K63Ub
70
GluA2
100
CYLD
100

Figure 4E

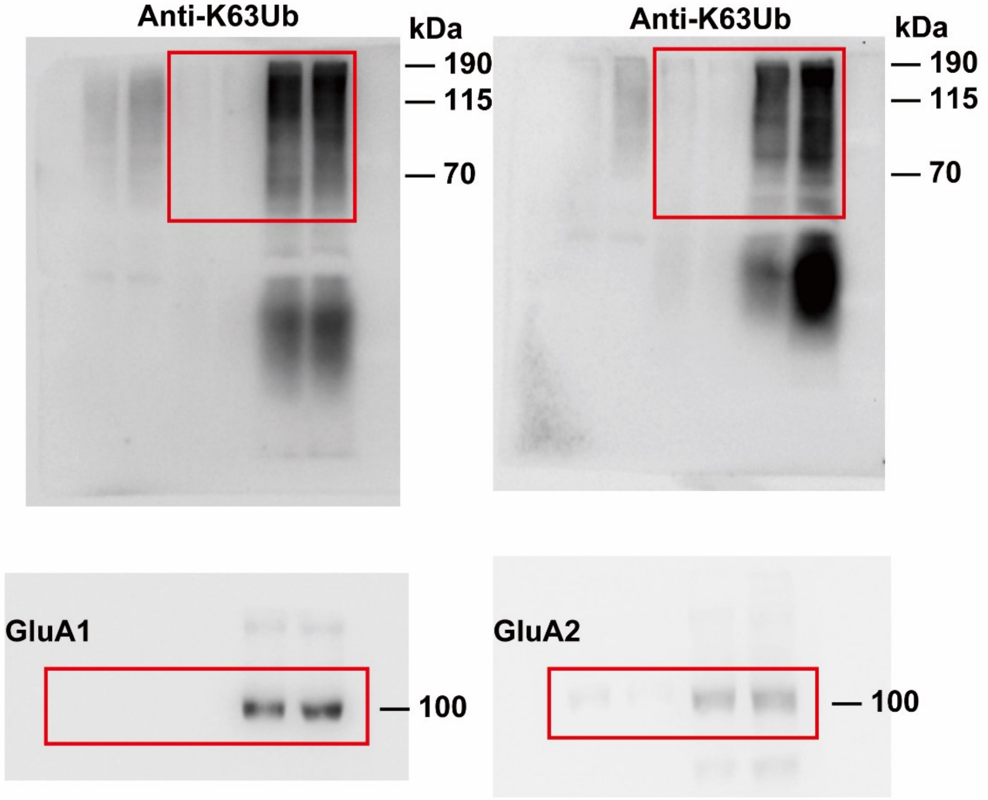

Figure 4G

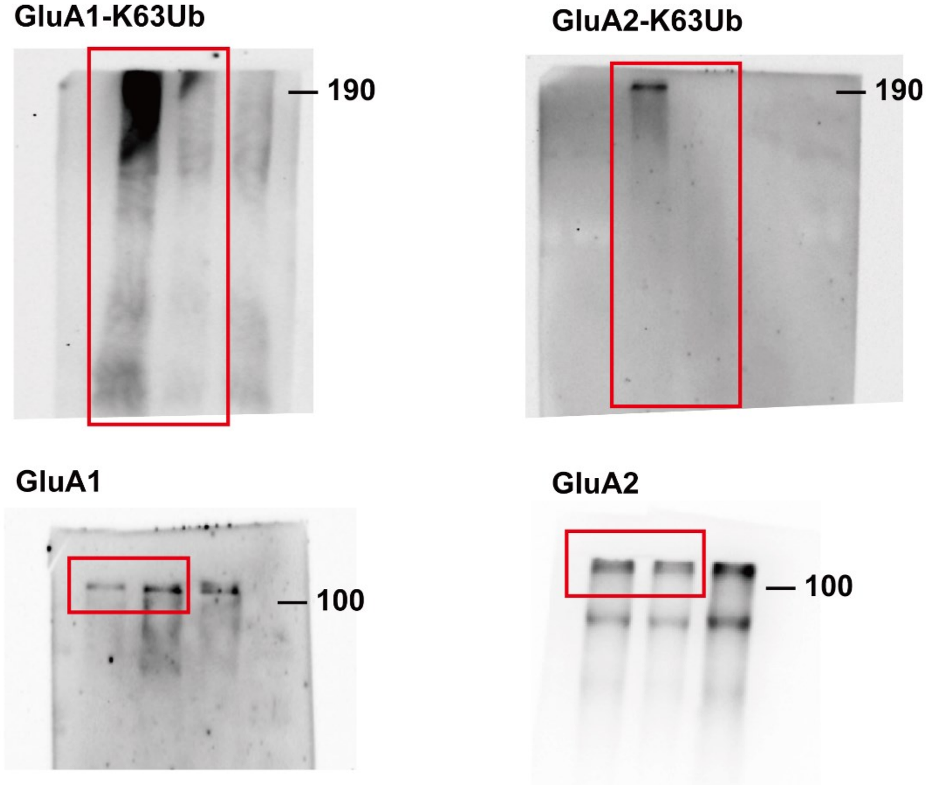

Supplement: Supplementary file 3 [file Data_Sheet_3.pdf]
